# Comprehensive characterization of complex glycosphingolipids in human pancreatic cancer tissues

**DOI:** 10.1016/j.jbc.2023.102923

**Published:** 2023-01-19

**Authors:** Karel Hořejší, Chunsheng Jin, Zuzana Vaňková, Robert Jirásko, Ondřej Strouhal, Bohuslav Melichar, Susann Teneberg, Michal Holčapek

**Affiliations:** 1 University of Pardubice, Faculty of Chemical Technology, Department of Analytical Chemistry, , Pardubice, Czech Republic; 2 University of South Bohemia in České Budějovice, Faculty of Science, Department of Chemistry, České Budějovice, Czech Republic; 3 University of Gothenburg, Sahlgrenska Academy, Proteomics Core Facility, Göteborg, Sweden; 4 Palacký University Olomouc, Faculty of Medicine and Dentistryand University Hospital, Department of Oncology, Olomouc, Czech Republic; 5 University of Gothenburg, Sahlgrenska Academy, Institute of Biomedicine, Department of Medical Biochemistry and Cell Biology, Göteborg, Sweden

**Keywords:** Lipidomics, lipids, monoclonal antibodies, glycolipids, sphingolipids, pancreatic cancer, Tandem mass spectrometry, liquid chromatography, thin-layer chromatography, chromatogram binding assay, A-GSL, acid GSL, BPC, base peak chromatogram, FUT, fucosyltransferase, GSL, glycosphingolipid, HILIC, hydrophilic interaction liquid chromatography, LC/ESI-MS^2^, liquid chromatography electrospray ionization tandem mass spectrometry, N-GSL, neutral GSL, PDAC, pancreatic ductal adenocarcinoma, rEGCase II, recombinant endoglycoceramidase II

## Abstract

Pancreatic ductal adenocarcinoma (PDAC) is one of the most common causes of cancer-related deaths worldwide, accounting for 90% of primary pancreatic tumors with an average 5-year survival rate of less than 10%. PDAC exhibits aggressive biology, which, together with late detection, results in most PDAC patients presenting with unresectable, locally advanced, or metastatic disease. In-depth lipid profiling and screening of potential biomarkers currently appear to be a promising approach for early detection of PDAC or other cancers. Here, we isolated and characterized complex glycosphingolipids (GSL) from normal and tumor pancreatic tissues of patients with PDAC using a combination of TLC, chemical staining, carbohydrate-recognized ligand-binding assay, and LC/ESI-MS^2^. The major neutral GSL identified were GSL with the terminal blood groups A, B, H, Le^a^, Le^b^, Le^x^, Le^y^, P1, and PX2 determinants together with globo- (Gb_3_ and Gb_4_) and neolacto-series GSL (nLc_4_ and nLc_6_). We also revealed that the neutral GSL profiles and their relative amounts differ between normal and tumor tissues. Additionally, the normal and tumor pancreatic tissues differ in type 1/2 core chains. Sulfatides and GM_3_ gangliosides were the predominant acidic GSL along with the minor sialyl-nLc_4_/nLc_6_ and sialyl-Le^a^/Le^x^. The comprehensive analysis of GSL in human PDAC tissues extends the GSL coverage and provides an important platform for further studies of GSL alterations; therefore, it could contribute to the development of new biomarkers and therapeutic approaches.

Pancreatic ductal adenocarcinoma (PDAC) is the most prevalent type of primary pancreatic malignant tumors (accounting for more than 90% of all types of pancreatic cancer) with highly aggressive behavior and extremely poor prognosis (1, 2, 3). A major problem in the treatment of PDAC consists mainly of the difficult diagnosis of early stage (*i.e.*, T1 and T2 tumors), which are usually asymptomatic. Most patients (∼80%) are diagnosed in advanced stages (*i.e.*, T3 or T4 tumors with lymph node and distant metastases) and are not eligible for complete surgical resection and thus incurable (1, 4). Another significant hallmark of PDAC is high resistance and low response rate to treatment with anticancer drugs and radiation (1, 2, 5). The high resistance of PDAC to available therapies, together with late detection, results in a 5-year overall survival rate of less than 10% and, particularly in metastatic PDAC, an overall 1-year survival rate of less than 20%. This makes PDAC the most lethal cancer (1, 2, 3, 6). Therefore, novel diagnostic biomarkers for early cancer detection are urgently needed (2, 5).

The carbohydrate antigen sialyl Lewis^a^ (*i.e.*, sLe^a^ or CA 19-9) is one of the well-known and frequently used serological biomarkers for the clinical diagnosis of pancreatic (7, 8), gastrointestinal, and other types of epithelial cancers (9). The determination of CA 19-9 test is routinely used to monitor treatment response in patients with advanced PDAC. However, the limited sensitivity and specificity does not allow to use CA 19-9 as a diagnostic biomarker for early stage tumors since CA 19-9 concentrations do not increase in a substantial percentage of patients with PDAC, and increased levels may be observed in patients with non-neoplastic disorders, despite high specificity for high cutoff values. Consequently, the CA19-9 assay is of limited utility for the diagnosis or monitoring of PDAC, preventing its use for early detection (10, 11, 12, 13). In a recent paper by Wolrab *et al.* (14), it was concluded that MS-based lipidomic profiling of human blood outperforms common clinical methods established for the monitoring of PDAC progression, including the CA 19-9 test.

Lipids have several key functions in human metabolism, such as constituting cell membrane components, signal molecules, energy supply, storage, and barriers (15, 16, 17). Specifically, glycosphingolipids (GSL) are ubiquitous constituents of eukaryotic plasma membranes and membrane-bound subcellular organelles that occur along with the most abundant phospholipids (15, 18, 19). GSL consist of a hydrophobic ceramide backbone bound to a hydrophilic carbohydrate part by a glycosidic bond, and both parts show immense structural diversity that makes them remarkably assorted compounds (18). Furthermore, GSL with blood group determinants is well known to be synthesized at high levels in the pancreas (20). Aberrant expression of GSL including alterations in the composition and concentrations of GSL and lipids is a typical hallmark of a wide range of cancers (7, 14, 21, 22, 23, 24, 25), which has been extensively documented in cancer cell lines (22, 26, 27, 28, 29) or tissues (20, 24, 30, 31, 32, 33, 34) and also reported in body fluids of cancer patients (35, 36, 37, 38). Several of the studies mentioned above concluded that the reported dysregulation of lipid metabolism in cancer cells is relevant to distinguish cancer patients from healthy controls, suggesting that changes in lipidomes are strongly associated with cancer progression (6).

Glycosylation occurs in all organisms and plays a crucial role in many cellular processes (39, 40, 41, 42). The disruption of glycosylation, such as aberrant glycan structure formation and alteration of glycosylation pathways, is probably intricately associated with a number of disorders including malignant transformation and tumor progression (19, 40, 42, 43). This may also be accompanied by the expression of tumor-associated carbohydrate antigens (39). As a consequence, changes in lipid metabolism and glycosylation have received significant attention in recent decades and are commonly documented in cancer investigations (40). Alterations in glycan structures have been observed in many cancers (42, 44, 45). However, the complex biology of cancer development and progression is not yet fully understood. Investigations are specifically aimed at pathways linked to two main types of protein glycosylation, that is, N-linked and O-linked glycosylation, to reveal its role in cancer pathogenesis (39). Moreover, the results obtained by Zhang *et al.* (6) demonstrated that GSL-glycosylation and O-glycosylation play a more dominant role, in particular in pancreatic cancer, than N-glycosylation (46). However, targeted approaches that focus mainly on tumor cells and predefined metabolic pathways may not show the full extent of complex metabolic alterations (5). In addition, there are still major challenges that stem mainly from the lack of sensitive, accurate, and reliable methods for the separation of GSL isomers as well as for the detection, identification, and quantitation of less prevalent GSL species (47).

The aim of the present study is to characterize the GSL of human pancreatic tissues of patients with PDAC with a particular interest in minor complex GSL to expand the database of lipids that are routinely analyzed and to allow mutual comparison of GSL alterations in normal and tumor pancreatic tissues. The future perspective of this study is to incorporate these complex GSL into the screening method for PDAC based on body fluid analysis, as recently published by our research group (14).

## Results

### Isolation of GSL for in-depth analysis

**Figure 1:**
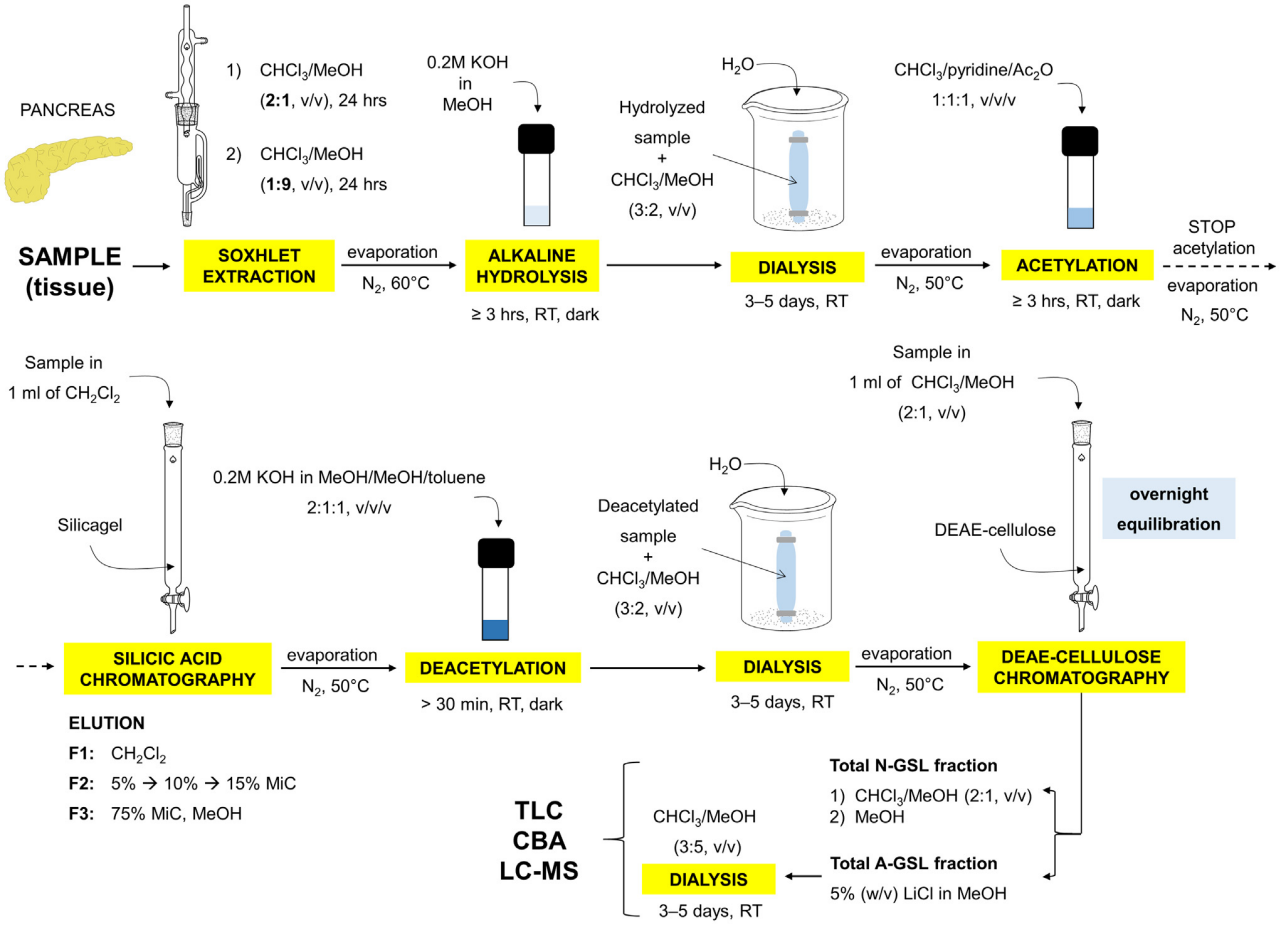
**Schematic representation of a protocol for the isolation of glycosphingolipids.** RT denotes room temperature, Ac_2_O denotes acetic anhydride, MiC denotes methanol in chloroform, TLC denotes thin-layer chromatography, CBA denotes chromatogram-binding assay, LC-MS denotes liquid chromatography-mass spectrometry.

The GSL were isolated by a micro method (Fig. 1) according to Barone *et al.* (48), which allows the isolation and purification of GSL with a wider range of carbohydrate units. This is of particular advantage for complex GSL that are found in biological materials in tiny amounts, and their effective isolation by conventional extraction methods, such as Folch (49), Bligh and Dyer (50), or Matyash (51), has not yet been described.

In total, 24 paired tissue samples of tumor and normal tissues were collected from 12 patients. After total lipid extraction, the extracts were subjected to mild alkaline methanolysis to remove acylglycerols and alkali-labile phospholipids. The purpose of the ensuing acetylation was to change the polarity of glycolipids from polar to nonpolar so that alkali-stable phospholipids (mainly sphingomyelins) were removed. Consequently, acetylated GSL were separated from the nonpolar compounds (*e.g.*, ceramides) and alkali-stable phospholipids (especially sphingomyelins) using silica-based chromatography. After deacetylation, the GSL were separated into neutral GSL (N-GSL) and acid GSL (A-GSL) fractions using ion-exchange chromatography. In summary, 6.3 mg and 26.2 mg of N-GSL were obtained, together with 11.6 mg and 14.3 mg of A-GSL from pooled tumor and normal pancreatic tissues, respectively (Table 1).

**Table 1:** Amounts of acid and neutral glycosphingolipids obtained from normal and tumor pancreatic tissues of PDAC patients and expressed in mg of glycosphingolipids per g of tissues in dry weight

Type of sample	Wet weight [g]	Dry weight [g]	N-GSL [mg]	N-GSL [mg/g tissue]	A-GSL [mg]	A-GSL [mg/g tissue]
Pooled tissues; T	1.089	0.606	6.3	10.4	11.6	19.1
Pooled tissues; N	2.092	1.232	26.2	21.3	14.3	11.6

N-GSL and A-GSL denote total neutral and acid glycosphingolipids, respectively. T and N denote tumor and normal, respectively, and ND denotes not determined.

*Rhodococcus* spp. recombinant endoglycoceramidase II (rEGCase II) was used for the hydrolysis of GSL, although the hydrolytic capacity of this enzyme to globo-series GSL and some gangliosides is restricted (28). In contrast, EGCase I has a broader substrate specificity and better reaction efficiency than EGCase II and III (52, 53). However, the use of rEGCase II in this study was intentional because globotriaosylceramide and globotetraosylceramide (Gb_3_ and Gb_4_) are major GSL of many tissues, resulting in MS spectra being dominated by Gb_3_ and Gb_4_ ions. The main advantage of using rEGCase II in this study is that it allowed the detection of low abundant complex GSL.

### Separation and structural characterization of GSL

We performed liquid chromatography electrospray ionization tandem mass spectrometry (LC/ESI-MS^2^) analysis of intact GSL (both N- and A-GSL) and neutral GSL-derived oligosaccharides from human pancreatic cancer and surrounding normal tissues. The major mono- and di-hexosylceramides (*i.e.*, GlcCer, GalCer, LacCer), globotriaosylceramides and globotetraosylceramides (*i.e.*, Gb_3_ and Gb_4_), and (neo)lacto-GSL together with several ganglioside subclasses and sulfatides have been extensively investigated in various biological matrices, as thoroughly summarized in studies by Zhuo *et al.* (54) and Wolrab *et al.* (23). In contrast, only a few recent studies showed altered complex GSL in most tumor cells (6, 33, 34). Therefore, this study focuses mainly on tetrasaccharides and larger oligosaccharides with the goal of comparing the GSL profiles of normal and tumor pancreatic tissues and implementing the GSL database for lipidomic analysis.

#### LC/ESI-MS^2^ of neutral GSL-derived oligosaccharides

**Figure 2:**
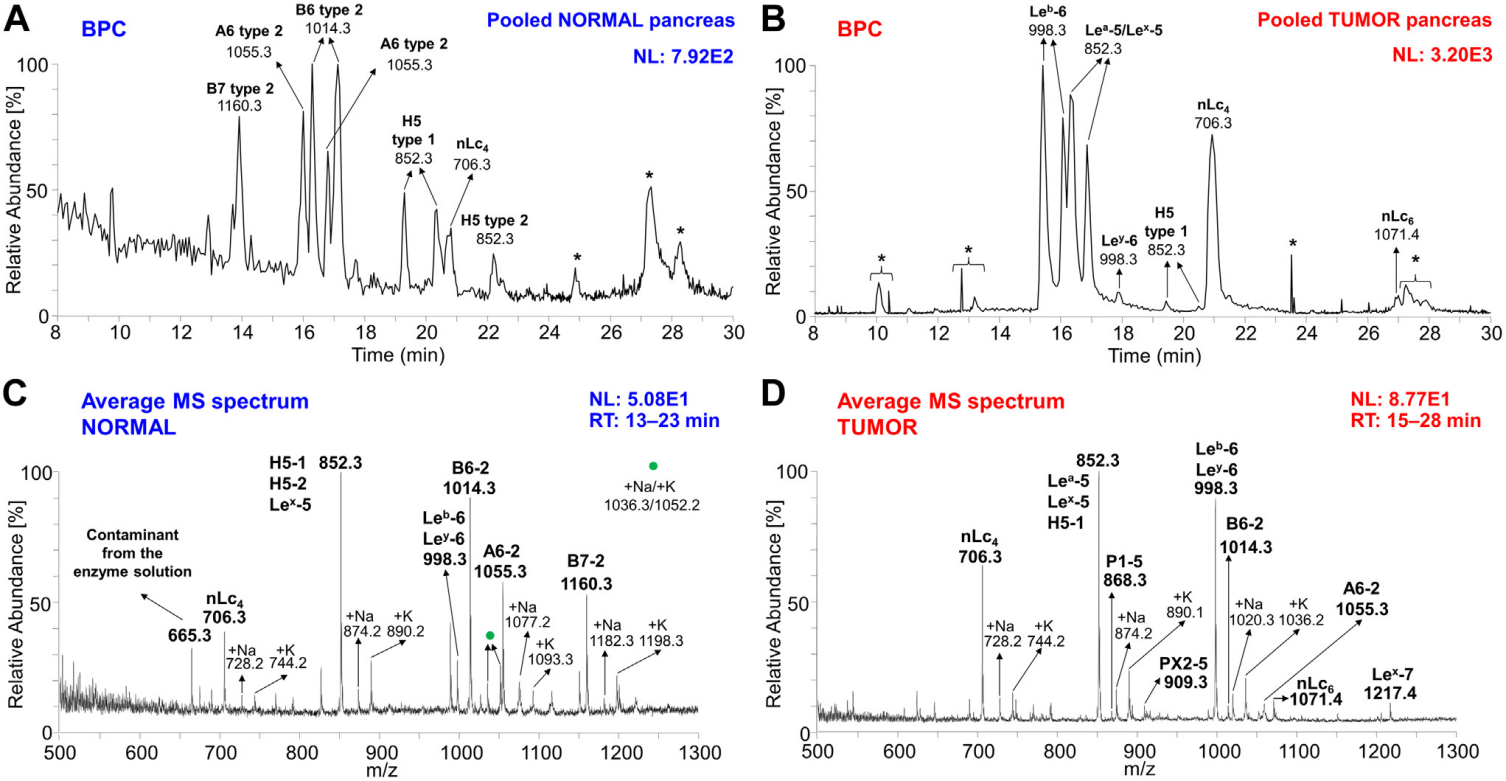
**Characterization of the oligosaccharides obtained from neutral glycosphingolipid fractions from pooled normal and tumor pancreatic tissues of patients suffering from pancreatic ductal adenocarcinoma, by hydrolysis with endoglycoceramidase II from*****Rhodococcus*****spp.** Base peak chromatogram (BPC) from LC/ESI-MS (negative ion mode at *m/z* 380–2000, retention time 8–30 min) of the neutral glycosphingolipid fraction obtained from pooled normal (*A*) and tumor (*B*) pancreatic tissues illustrating the predominant glycan chains. The average MS spectrum of the neutral glycosphingolipid fraction obtained from pooled normal (*C*) and tumor (*D*) pancreatic tissues. The identification of oligosaccharides was based on their retention times, determined molecular masses, and subsequent MS^2^ sequencing. The oligosaccharides identified in the chromatograms were as follows: B7-2, Galα3(Fucα2)Galβ4(Fucα3)GlcNAcβ3Galβ4Glc; B6-2, Galα3(Fucα2)Galβ4GlcNAcβ3Galβ4Glc; A6-2, GalNAcα3(Fucα2)Galβ4GlcNAcβ3Galβ4Glc; Le^a^-5, Galβ3(Fucα4)GlcNAcβ3Galβ4Glc; Le^x^-5, Galβ4(Fucα3)GlcNAcβ3Galβ4Glc; Le^b^-6, Fucα2Galβ3(Fucα4)GlcNAcβ3Galβ4Glc; Le^y^-6, Fucα2Galβ4(Fucα3)GlcNAcβ3Galβ4Glc; H5-1, Fucα2Galβ3GlcNAcβ3Galβ4Glc; H5-2, Fucα2Galβ4GlcNAcβ3Galβ4Glc; nLc_4_, Galβ4GlcNAcβ3Galβ4Glc; nLc_6,_ Galβ4GlcNAcβ3Galβ4GlcNAcβ3Galβ4Glc; Le^x^-7, Galβ4(Fucα3)GlcNAcβ3Galβ4GlcNAcβ3Galβ4Glc; PX2-5, GalNAcβ3Galβ4GlcNAcβ3Galβ4Glc; and P1-5, Galα4Galβ4GlcNAcβ3Galβ4Glc. NL denotes the normalization level (*i.e.*, intensity of the most abundant peak), RT denotes retention time, ∗ denotes nonglycolipid contaminant.

Oligosaccharides released from total N-GSL fractions isolated from the tumor and surrounding normal tissues were analyzed by LC/ESI-MS^2^ in the negative-ion mode (Fig. 2).

**Figure 3:**
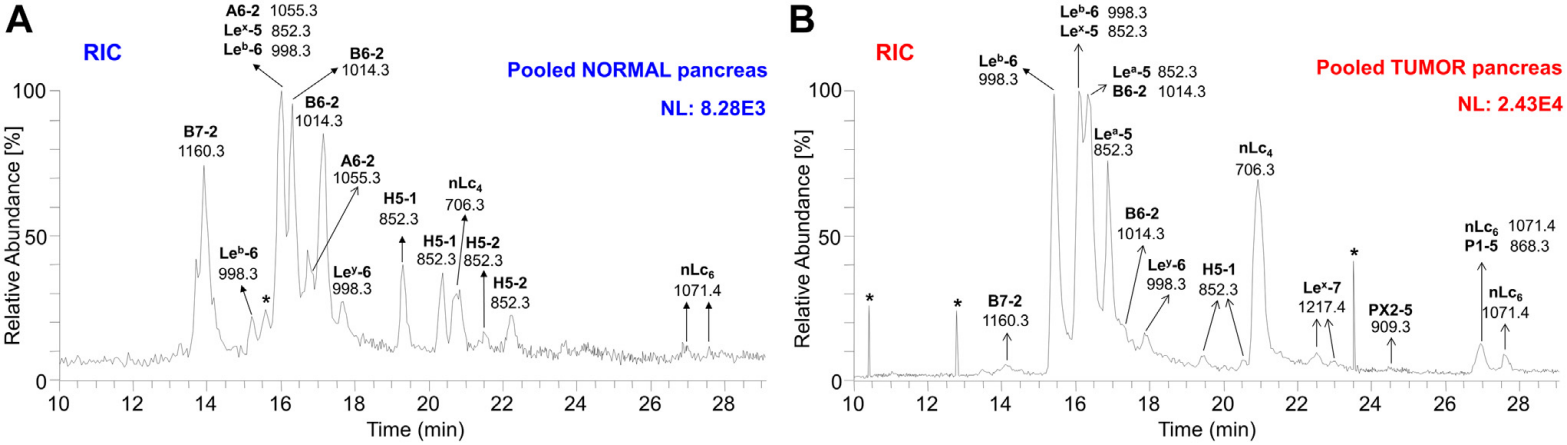
**Characterization of theoligosaccharides obtained from neutral glycosphingolipid fractions from pooled normal and tumor pancreatic tissues of patients suffering from pancreatic ductal adenocarcinoma, by hydrolysis with endoglycoceramidase II from*****Rhodococcus spp*****.** Reconstructed ion current (RIC) chromatogram from LC/ESI-MS (negative ion mode at *m/z* 500–1300, retention time 10–29 min) of the neutral glycosphingolipid (GSL) fraction obtained from pooled normal (*A*) and tumor (*B*) pancreatic tissues depicting all identified and confirmed GSL subclasses. The identification of oligosaccharides was based on their retention times, determined molecular masses, and subsequent MS^2^ sequencing. The oligosaccharides identified in the chromatograms were as follows: B7-2, Galα3(Fucα2)Galβ4(Fucα3)GlcNAcβ3Galβ4Glc; B6-2, Galα3(Fucα2)Galβ4GlcNAcβ3Galβ4Glc; A6-2, GalNAcα3(Fucα2)Galβ4GlcNAcβ3Galβ4Glc; Le^a^-5, Galβ3(Fucα4)GlcNAcβ3Galβ4Glc; Le^x^-5, Galβ4(Fucα3)GlcNAcβ3Galβ4Glc; Le^b^-6, Fucα2Galβ3(Fucα4)GlcNAcβ3Galβ4Glc; Le^y^-6, Fucα2Galβ4(Fucα3)GlcNAcβ3Galβ4Glc; H5-1, Fucα2Galβ3GlcNAcβ3Galβ4Glc; H5-2, Fucα2Galβ4GlcNAcβ3Galβ4Glc; nLc_4_, Galβ4GlcNAcβ3Galβ4Glc; nLc_6,_ Galβ4GlcNAcβ3Galβ4GlcNAcβ3Galβ4Glc; Le^x^-7, Galβ4(Fucα3)GlcNAcβ3Galβ4GlcNAcβ3Galβ4Glc; PX2-5, GalNAcβ3Galβ4GlcNAcβ3Galβ4Glc; and P1-5, Galα4Galβ4GlcNAcβ3Galβ4Glc. NL denotes normalization level (*i.e.*, intensity of the most abundant peak), RT denotes retention time, ∗ denotes nonglycolipid contaminant.

Base peak chromatograms (BPCs) from pooled normal (Fig. 2*A*) and tumor pancreatic tissues (Fig. 2*B*) revealed that the most intense peaks, corresponding to the respective oligosaccharide chains, differed significantly. The most predominant ions detected as [M–H]^–^ in pooled normal pancreas tissues (Fig. 2*A*) were ions at *m/z* 1160.3, 1014.3, and 1055.3, while in the pooled pancreatic tumor tissues (Fig. 2*B*), the dominant ions were at *m/z* 998.3, 852.3, and 706.3, To inspect the composition of both types of tissues in depth, we constructed average MS spectra of deprotonated molecules (Fig. 2, *C* and *D*) and the reconstructed ion current chromatograms (Fig. 3, *A* and *B*). Reconstructed ion current chromatograms were constructed by extracting all *m/z* values corresponding to the analytes of interest. The ion profiles of deprotonated molecules show a number of lipids detected as [M–H]^–^, ranging mainly from tetrasaccharides to heptasaccharides (Fig. 2, *C* and *D*).

**Figure 4:**
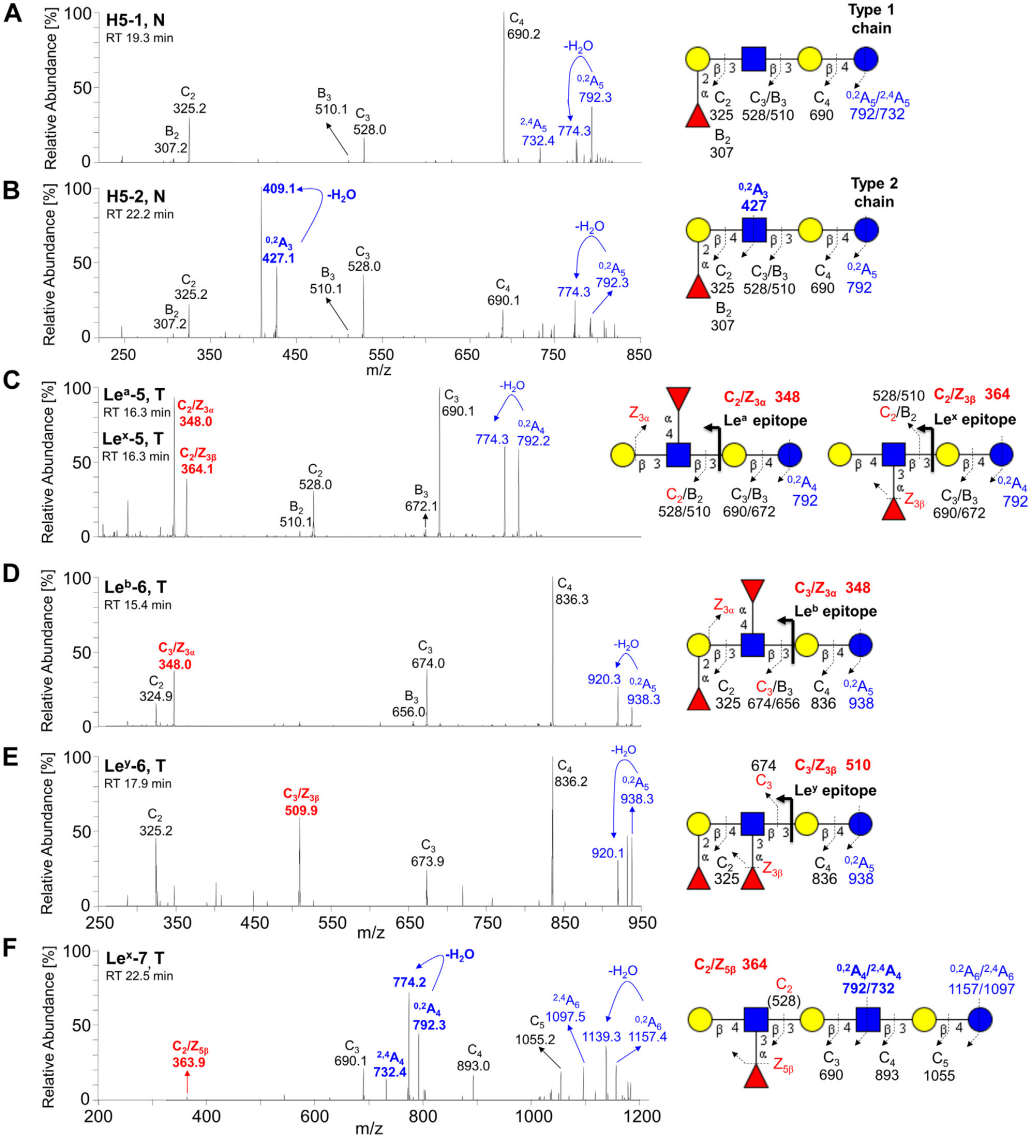
**LC/ESI-MS**^**2**^**characterization of the neutral oligosaccharides obtained by hydrolysis with endoglycoceramidase II from *Rhodococcus spp.* from pooled normal and tumor pancreatic tissues of patients with pancreatic ductal adenocarcinoma together with the respective interpretation formulas – part I.** MS^2^ spectra of the molecular ions at *m/z* 852.3 at retention time (*A*) 19.3 min (*i.e.*, H5-1), (*B*) 22.2 min (*i.e.*, H5-2), (*C*) 16.3 min (*i.e.*, Le^a^-5 and Le^x^-5). MS^2^ spectra of the molecular ions at *m/z* 998.3 at retention time (*D*) 15.4 min (*i.e.*, Le^b^-6) and (*E*) 17.9 min (*i.e.*, Le^y^-6). MS^2^ spectrum of the molecular ion at *m/z* 1217.4 at retention time (*F*) 22.5 min (*i.e.*, Le^x^-7). The identification of oligosaccharides was based on their retention times, determined molecular masses, and subsequent MS^2^ sequencing. T denotes tumor tissue, N denotes normal tissues, and RT denotes retention time. LC/ESI-MS^2^, liquid chromatography electrospray ionization tandem mass spectrometry.

**Figure 5:**
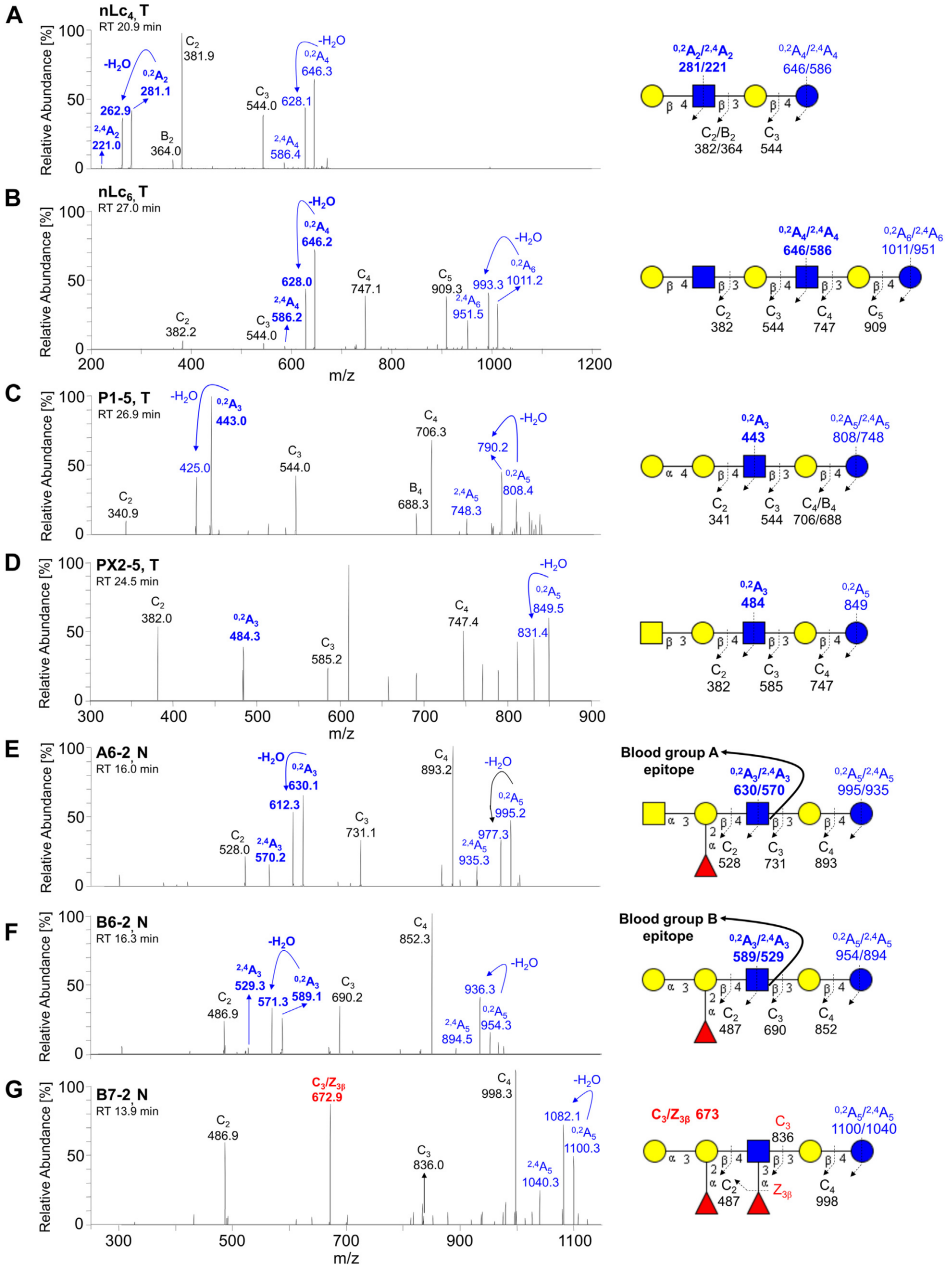
**LC/ESI-MS**^**2**^**characterization of the neutral oligosaccharides obtained by hydrolysis with endoglycoceramidase II from *Rhodococcus spp.* from pooled normal and tumor pancreatic tissues of patients with pancreatic ductal adenocarcinoma together with the respective interpretation formulas – part II.** MS^2^ spectrum of the molecular ion at (*A*) *m/z* 706.3 at retention time 20.9 min (*i.e.*, nLc_4_), (*B*) *m/z* 1071.4 at retention time 27.0 min (*i.e.*, nLc_6_), (*C*) *m/z* 868.3 at retention time 26.9 min (*i.e.*, P1-5), (*D*) *m/z* 909.3 at retention time 24.5 min (*i.e.*, PX2-5), (*E*) *m/z* 1055.4 at retention time 16.0 min (*i.e.*, A6-2), (*F*) *m/z* 1014.3 at retention time 16.3 min (*i.e.*, B6-2), and (*G*) *m/z* 1160.3 at retention time 13.9 min (*i.e.*, B7-2). The identification of oligosaccharides was based on their retention times, determined molecular masses, and subsequent MS_2_ sequencing. T denotes tumor tissue, N denotes normal tissues, and RT denotes retention time. LC/ESI-MS^2^, liquid chromatography electrospray ionization tandem mass spectrometry.

The subsequent MS^2^ of these ions (as exemplified in Figs. 4 and 5) identified neolacto tetrasaccharides (nLc_4_, *m/z* 706.3), neolacto hexasaccharides (nLc_6_, *m/z* 1071.4), P1 pentasaccharides (P1-5, *m/z* 868.3), PX2 pentasaccharides (PX2-5, *m/z* 909.3), H type 1 and 2 and Le^a^/Le^x^ pentasaccharides (H5-1 and 2, Le^a/x^-5, *m/z* 852.3), Le^b^/Le^y^ hexasaccharides (Le^b/y^-6, *m/z* 998.3), Le^x^ heptasaccharides (Le^x^-7, *m/z* 1217.4), the blood group A type 2 hexasaccharides (A6-2, *m/z* 1055.3), the blood group B type 2 hexasaccharides (B6-2, *m/z* 1014.3), and the blood group B type 2 heptasaccharides (B7-2, *m/z* 1160.3).

Most of these observed deprotonated molecules, particularly those most abundant, were additionally confirmed by the presence of sodium and potassium adducts (*i.e.*, [M–2H+Na]^–^ and [M–2H + K]^–^) in ion profiles of deprotonated molecules, as depicted in Figure 2, *C* and *D*. A detailed interpretation of MS^2^ spectra of individual oligosaccharides identified in the samples (Figs. 4 and 5) is described below.

##### H type 1 and H type 2 pentasaccharides

**Figure 6:**
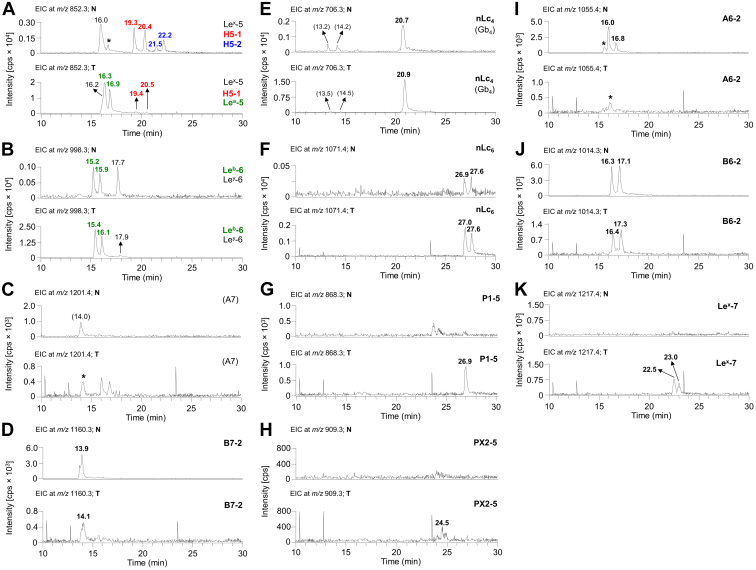
**Extracted ion current chromatograms of the neutral glycosphingolipid fractions isolated by treatment with endoglycoceramidase II (*Rhodococcus spp.*) and obtained from pooled normal and tumor pancreatic tissues of patients with pancreatic ductal adenocarcinoma.***A*, EIC at *m/z* 852.3 (H type 1/2, Le^a^, and Le^x^ pentasaccharides), (*B*) EIC at *m/z* 998.3 (Le^b^ and Le^y^ hexasaccharides), (*C*) EIC at *m/z* 1201.4 (blood group A heptasaccharides), (*D*) EIC at *m/z* 1160.3 (blood group B type 2 heptasaccharides), (*E*) EIC at *m/z* 706.3 (neolactotetrasaccharides and globotetrasaccharides), (*F*) EIC at *m/z* 1071.4 (neolactohexasaccharides), (*G*) EIC at *m/z* 868.3 (P1 pentasaccharides), (*H*) EIC at *m/z* 909.3 (PX2 pentasaccharides), (*I*) EIC at *m/z* 1055.4 (blood group A type 2 hexasaccharides), (*J*) EIC at *m/z* 1014.3 (blood group B type 2 hexasaccharides), (*K*) EIC at *m/z* 1217.4 (Le^x^ heptasaccharides). The identification of oligosaccharides was based on their retention times, determined molecular masses, and subsequent MS^2^ sequencing. N denotes normal tissues, T denotes tumor tissue, and ∗ denotes nonglycolipid contaminants.

Oligosaccharides derived from the H type 1 (*i.e.*, H type 1 penta) and H type 2 (*i.e.*, H type 2 penta) glycosylceramides were analyzed by LC-MS^2^. MS^2^ spectra of the ions at *m/z* 852.3 eluted at 19.3 min (H type 1 penta, Fig. 4*A*) and 22.2 min (H type 2 penta, Fig. 4*B*) in both cases resulted in a series of prominent C-type fragment ions (C_2_ at *m/z* 325.2, C_3_ at *m/z* 528.0, and C_4_ at *m/z* 690.2) together with low abundant B-type fragments (B_2_ at *m/z* 307.2 and B_3_ at *m/z* 510.1) arising from additional loss of water from the corresponding C-type fragment ions. This sequence of fragment ions identifies the pentasaccharide with Fuc-Hex-HexNAc-Hex-Hex sequence. Furthermore, the fragment ions ^0,2^A_5_/^0,2^A_5_-H_2_O at *m/z* 792.3/774.3 and ^2,4^A_5_ at *m/z* 732.4 were assigned as the cross-ring glucose cleavage at the reducing end. The H type 1 penta and H type 2 penta core chains were distinguished based on the diagnostic fragment ions ^0,2^A_3_/^0,2^A_3_-H_2_O at *m/z* 427.1/409.1 for H type 2 (55, 56). Taken together, this allows the identification of H type 1 pentasaccharide (Fucα2Galβ3GlcNAcβ3Galβ4Glc) and H type 2 pentasaccharide (Fucα2Galβ4GlcNAcβ3Galβ4Glc). The H type 1 pentasaccharide was detected in both pooled tumor and normal tissues, while the H type 2 pentasaccharide was observed only in pooled normal tissues (Figs. 3, *A* and *B* and 6*A*).

##### Lewis^a^ and Lewis^x^ pentasaccharides

MS^2^ spectra of the isomeric ions of protonated molecules at *m/z* 852.3 eluted at 16.3 min (Figs. 4*C*, and 6*A*) resulted in a series of C-/B-type fragment ions (C_2_ at *m/z* 528.0, B_2_ at *m/z* 510.1, C_3_ at *m/z* 690.1, and B_3_ at *m/z* 672.1) alongside the fragment ions ^0,2^A_4_ at *m/z* 792.2 and ^0,2^A_4_-H_2_O at *m/z* 774.3 defining the oligosaccharide sequence as Hex(Fuc)HexNAc-Hex-Hex. Two double cleavage ions at *m/z* 348.0 and 364.1 were observed, which were derived from C/Z cleavage of internal GlcNAc. According to MS^2^ in the spectrum of the LNFP II/III standard and the study published by Chai *et al.* (56), these were diagnostic ions for Le^a^ pentasaccharide (Le^a^-5, *m/z* 348.0) and Le^x^ pentasaccharide (Le^x^-5, *m/z* 364.1). Thus, Le^a^-5 and Le^x^-5 were coeluted with overlapped MS^2^ spectra. Taken together, these features identified the Le^a^ pentasaccharide (Galβ3(Fucα4)GlcNAcβ3Galβ4Glc) and Le^x^ pentasaccharide (Galβ4(Fucα3)GlcNAcβ3Galβ4Glc). Le^a^-5 was observed only in pooled tumor tissue (eluted at 16.3 and 16.9 min, Figs. 3, *A* and *B* and 6*A*), while Le^x^-5 was found in both pooled normal and tumor tissues (eluted at 16.0 and 16.2 min, respectively, Figs. 3, *A* and *B* and 6*A*).

##### Lewis^b^ and Lewis^y^ hexasaccharides

LC-MS^2^ of [M–H]^–^ ions at *m/z* 998.3 revealed hexasaccharides with a composition of Hex_3_HexNAc_1_Fuc_2_ eluting at 15.4 and 17.9 min, respectively. MS^2^ spectra of the ions at *m/z* 998.3 defined Le^b^ hexa at 15.4 min (Fig. 4*D*) and Le^y^ hexa at 17.9 min (Fig. 4*E*) and were characterized by a series of prominent C-type fragment ions (C_2_ at *m/z* 325.2, C_3_ at *m/z* 674.0, and C_4_ at *m/z* 836.2) and fragment ions ^0,2^A_5_ at *m/z* 938.3 and ^0,2^A_5_-H_2_O at *m/z* 920.1 formed by a cross-ring cleavage of a glucose at the reducing end indicating a terminal Le^b/y^. The linkage positions of internal GlcNAc substituted with fucose were identified by the diagnostic fragment ions resulting from the double glycosidic cleavage of the 3-linked oligosaccharide branch (*i.e.*, C_3_/Z_3α_ at *m/z* 348.0 for Le^b^ and C_3_/Z_3β_ at *m/z* 509.9 for Le^y^, Fig. 4, *D* and *E*, respectively) (56). These features identified the Le^b^ (Fucα2Galβ3(Fucα4)GlcNAcβ3Galβ4Glc) and Le^y^ (Fucα2Galβ4(Fucα3)GlcNAcβ3Galβ4Glc) hexasaccharides. Le^b^ and Le^y^ hexasaccharides were observed in pooled normal tissue (Fig. 3*A*) eluted at 15.2/15.9 min (Le^b^-6, Fig. 6*B*) and 17.7 min (Le^y^-6, Fig. 6*B*) and pooled tumor tissue (Fig. 3*B*) eluted at 15.4/16.1 min (Le^b^-6, Fig. 6*B*) and 17.9 min (Le^y^-6, Fig. 6*B*).

##### Lewis^x^ heptasaccharides

Additionally, we identified Le^x^ heptasaccharide (*i.e.*, Le^x^-7) by MS^2^ of the [M–H]^–^ ion at *m/z* 1217.4 eluted at 22.5 and 23.0 min (Fig. 6*K*). This minor ion was only observed in the pooled tumor tissue (Fig. 3*B*). The sequence Galβ4(Fucα3)GlcNAcβ3Galβ4GlcNAcβ3Galβ4Glc was confirmed by typical C-type fragment ions (C_3_ at *m/z* 690.1, C_4_ at *m/z* 893.0, and C_5_ at *m/z* 1055.2; C_2_ was missing) alongside the ions derived from the cross-ring cleavage of the glucose at the reducing end, *i.e.*, ^0,2^A_6_ at *m/z* 1157.4, ^0,2^A_6_-H_2_O at *m/z* 1139.3, and ^2,4^A_6_ at *m/z* 1097.5. The ions ^0,2^A_4_ at *m/z* 792.3, ^0,2^A_4_-H_2_O at *m/z* 774.2, and ^2,4^A_4_ at *m/z* 732.4 endorsed the presence of the innermost 4-substituted GlcNAc and together with the diagnostic fragment ion C_2_/Z_5β_ at *m/z* 363.9 affirming the terminal 3-linked branch of Gal-GlcNAc with Fuc at 3-position (Fig. 4*F*).

##### Neolacto series

LC-MS^2^ of [M–H]^–^ ions at *m/z* 706.3 and *m/z* 1071.4 identified saccharides with Hex_3_HexNAc and Hex_4_HexNAc_2_ composition, respectively. The glycan released with a composition of Hex_3_HexNAc that eluted at 20.7 and 20.9 min in both pooled normal and tumor tissues, respectively (Figs. 3, *A* and *B* and 6*E*), was characterized by MS^2^ of [M–H]^–^ ion at *m/z* 706.3 (Fig. 5*A*). The oligosaccharide Hex-HexNAc-Hex-Hex sequence was deduced from C-type fragment ion series (C_2_ at *m/z* 381.9 and C_3_ at *m/z* 544.0) along with low abundant fragment ions ^2,4^A_4_ at *m/z* 586.4, ^2,4^A_2_ at *m/z* 221.0, and B_2_ at *m/z* 364.0 and the notable ions ^0,2^A_4_ at *m/z* 646.3, ^0,2^A_4_-H_2_O at *m/z* 628.1, ^0,2^A_2_ at *m/z* 281.1, and ^0,2^A_2_-H_2_O at *m/z* 262.9. The latter two indicated a 4-substituted HexNAc, namely, type 2 chain (Galβ4GlcNAc). Together, MS^2^ indicates a neolacto tetrasaccharide (*i.e.*, nLc_4_, Galβ4GlcNAcβ3Galβ4Glc). Similarly, a glycan with a composition of Hex_4_HexNAc_2_ that eluted in both pooled normal and tumor tissues at 26.9/27.6 min and 27.0/27.6 min, respectively (Figs. 3, *A* and *B* and 6*F*), was characterized by MS^2^ of [M–H]^–^ ion at *m/z* 1071.4 (Fig. 5*B*). The MS^2^ spectra of the ions at *m/z* 706.3 and 1071.4 were very similar, however, in case of the ion at *m/z* 1071.4, the series of C-type fragment ions was extended by ions C_4_ at *m/z* 747.1 and C_5_ at *m/z* 909.3 together with ions ^0,2^A_6_ at *m/z* 1011.2, ^0,2^A_6_-H_2_O at *m/z* 993.3, and ^2,4^A_6_ at *m/z* 951.5 correlating with the additional Hex-HexNAc. Here, fragment ions ^0,2^A_4_ at *m/z* 646.2, ^0,2^A_4_-H_2_O at *m/z* 628.0, and ^2,4^A_4_ at *m/z* 586.2 corresponded with the innermost 4-substituted GlcNAc. Taken together, this identified neolactohexasaccharide (*i.e.*, nLc_6_, Galβ4GlcNAcβ3Galβ4GlcNAcβ3Galβ4Glc).

##### P1 and PX2 pentasaccharides

A pentasaccharide with the composition of Hex_4_HexNAc_1_ was observed only in the pooled tumor tissue and eluted at 26.9 min (Figs. 3*B* and 6*G*). The respective oligosaccharide sequence Hex-Hex-HexNAc-Hex-Hex was identified by the C-type fragment ions series (C_2_ at *m/z* 340.9, C_3_ at *m/z* 544.0, and C_4_ at *m/z* 706.3) alongside fragment ions ^0,2^A_5_ at *m/z* 808.4, ^0,2^A_5_-H_2_O at 790.2, and ^2,4^A_5_ at *m/z* 748.3, obtained by MS^2^ of the [M–H]^–^ ions at *m/z* 868.3 (Fig. 5*C*). Fragment ions ^0,2^A_3_ at *m/z* 443.0 and ^0,2^A_3_-H_2_O at *m/z* 425.0 were correlated with a 4-substituted internal GlcNAc. No information about the terminal Hex-Hex sequence was obtained by MS^3^. Based on the previous identification of P1 pentasaccharide (57) and considering that the α1,3-galactosyltransferase is not expressed in humans (58), we assumed that the features of the MS^2^ spectrum of [M–H]^–^ ions at *m/z* 868.3 allowed a tentative identification of the P1 pentasaccharide (Galα4Galβ4GlcNAcβ3Galβ4Glc).

A pentasaccharide with the composition of Hex_3_HexNAc_2_ was found only in the pooled tumor tissue and eluted at 24.5 min (Figs. 3*B* and 6*H*). The respective oligosaccharide HexNAc-Hex-HexNAc-Hex-Hex sequence was identified by the C-type fragment ions series (C_2_ at *m/z* 382.0, C_3_ at *m/z* 585.2, and C_4_ at *m/z* 747.4) together with ^0,2^A_5_ and ^0,2^A_5_-H_2_O ions at *m/z* 849.5 and 831.4, respectively, obtained by MS^2^ of the [M–H]^–^ ions at *m/z* 909.3 (Fig. 5*D*). Fragment ion ^0,2^A_3_ at *m/z* 484.3 was correlated with a 4-substituted internal GlcNAc; although the MS^2^ spectrum was very weak and did not completely allow a clear and safe interpretation of the oligosaccharide sequence, the MS^2^ spectrum was similar to the MS^2^ spectrum of the PX2 pentasaccharide identified by Westman *et al.* (59), which together with the identification of a relevant and very low abundant ion of protonated molecule at *m/z* 909.3 provides evidence suggesting the presence of the PX2 pentasaccharide (*i.e.*, GalNAcβ3Galβ4GlcNAcβ3Galβ4Glc).

##### Blood group A and B type 2 hexasaccharides and heptasaccharides

Deprotonated molecule [M–H]^–^ at *m/z* 1055.4 (Fig. 2*C*) consistent with the composition of Hex_3_HexNAc_2_Fuc_1_ was eluted only in pooled normal tissue at 16.0 and 16.8 min (Figs. 3*A* and 6*I*). The glycan sequence was deduced from the MS^2^ spectrum of the deprotonated molecule (Fig. 5*E*) based on a series of C-type fragment ions (C_2_ at *m/z* 528.0, C_3_ at *m/z* 731.1, and C_4_ at *m/z* 893.2) alongside the cross-ring fragment ions ^0,2^A_5_ at *m/z* 995.2, ^0,2^A_5_-H_2_O at *m/z* 977.3, and ^2,4^A_5_ at *m/z* 935.3. A type 2 core chain Galβ4GlcNAc was indicated by the fragment ions ^0,2^A_3_ at *m/z* 630.1, ^0,2^A_3_-H_2_O at *m/z* 612.3, and ^2,4^A_3_ at *m/z* 570.2. Thus, it was assigned as GalNAcα3(Fucα2)Galβ4GlcNAcβ3Galβ4Glc, *i.e*., a blood group A type 2 hexasaccharide (A6-2).

Deprotonated molecule [M–H]^–^ at *m/z* 1014.3 (Fig. 2, *C* and *D*) consistent with a composition of Hex_4_HexNAc_1_Fuc_1_ was eluted in both pooled normal and tumor tissue at 16.3/17.1 min (Figs. 3*A* and 6*J*) and 16.4/17.3 min (Figs. 3*B* and 6*J*), respectively. The oligosaccharide sequence was concluded from the series of C-type ions (C_2_ at *m/z* 486.9, C_3_ at *m/z* 690.2, and C_4_ at *m/z* 852.3) and cross-ring fragment ions ^0,2^A_5_ at *m/z* 954.3, ^0,2^A_5_-H_2_O at *m/z* 936.3, and ^2,4^A_5_ at *m/z* 894.5 of the MS^2^ spectrum (Fig. 5*F*). A type 2 core chain Galβ4GlcNAc was inferred from the fragment ions ^0,2^A_3_ at *m/z* 589.1, ^0,2^A_3_-H_2_O at *m/z* 571.3, and ^2,4^A_3_ at *m/z* 529.3. Taken together, it was assigned as Galcα3(Fucα2)Galβ4GlcNAcβ3Galβ4Glc, *i.e*., a blood group B group type 2 hexasaccharide (B6-2).

Deprotonated molecule [M–H]^–^ at *m/z* 1160.3 consistent with the composition of Hex_4_HexNAc_1_Fuc_2_ was eluted in both pooled normal and tumor tissues at 13.9 min and 14.1 min (Figs. 3, *A* and *B* and 6*D*), respectively. The oligosaccharide sequence was concluded from the MS^2^ spectrum (Fig. 5*G*) based on the series of C-type fragment ions (C_2_ at *m/z* 486.9, C_3_ at *m/z* 836.0, and C_4_ at *m/z* 998.3) together with cross-ring fragment ions ^0,2^A_5_ at *m/z* 1100.3, ^0,2^A_5_-H_2_O at *m/z* 1082.1, and ^2,4^A_5_ at *m/z* 1040.3. The diagnostic fragment ion C_3_/Z_3β_ at *m/z* 672.9 provided the evidence of 4-substituted GlcNAc with Fuc at 3-position and, furthermore, affirms the terminal 3-linked branch of GalGal(Fuc)GlcNAc. Therefore, it was assigned as Galcα3(Fucα2)Galβ4(Fucα3)GlcNAcβ3Galβ4Glc, *i.e.*, a blood group B type 2 heptasaccharide (B7-2).

**Figure 7:**
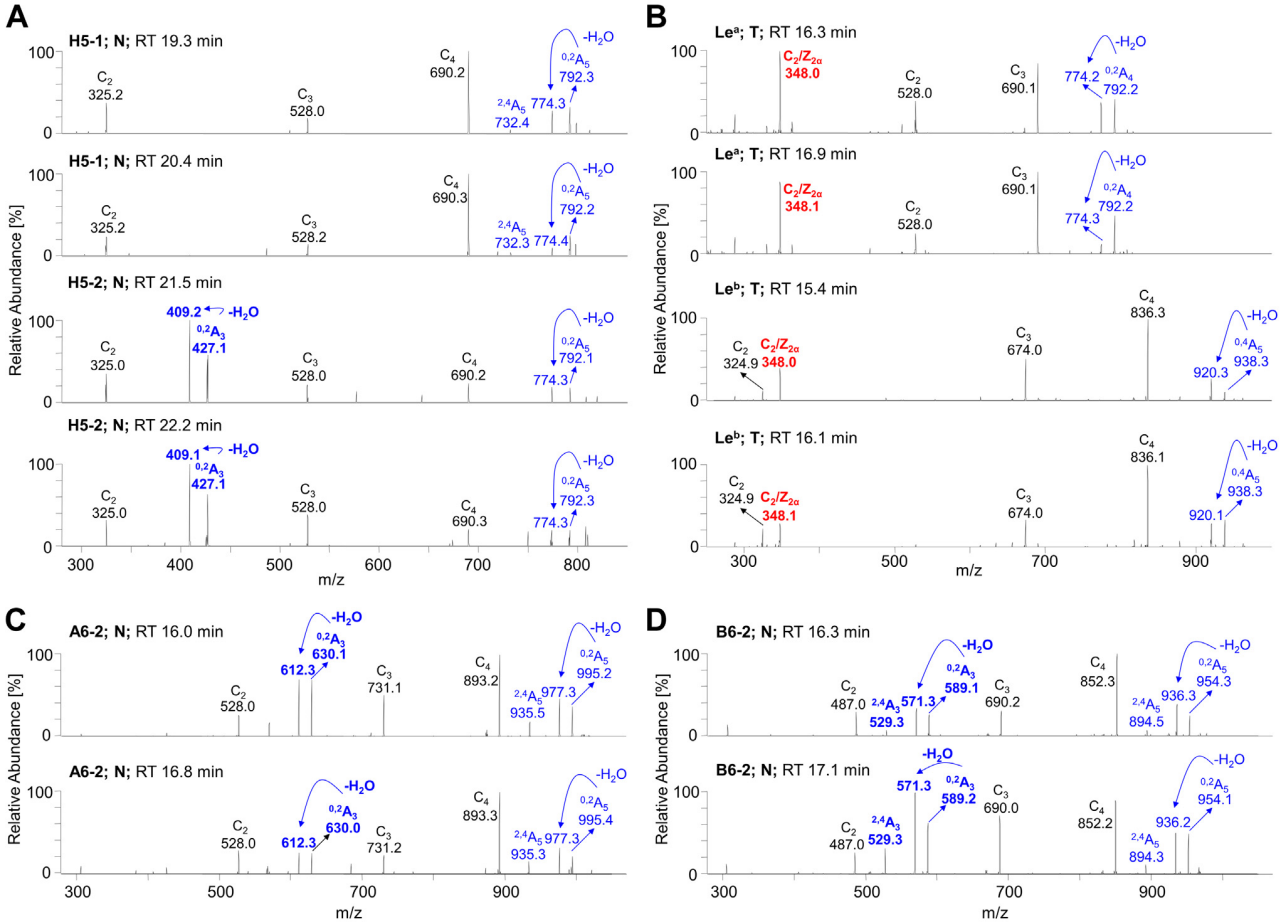
**Examples of MS**^**2**^**spectra of neutral glycosphingolipid observed as double peaks and isolated by treatment with endoglycoceramidase II from human normal and tumor pancreas.** MS^2^ spectra of double peaks with dominant deprotonated molecules (*A*) at *m/z* 852.3 confirmed the presence of H type 1 and H type 2 pentasaccharides (H5-1 and H5-2, respectively), (*B*) at *m/z* 852.3 and *m/z* 998.3 confirmed the presence of Le^a^ pentasaccharides (Le^a^-5) and Le^b^ hexasaccharides (Le^b^-6), respectively, (*C*) at *m/z* 1055.4 confirmed the presence of A type 2 hexasaccharides (A6-2), and (*D*) at *m/z* 1014.3 confirmed the presence of B type 2 hexasaccharides (B6-2). The identification of oligosaccharides was based on their retention times, determined molecular masses, and subsequent MS^2^ sequencing. N denotes normal tissues, T denotes tumor tissue, and RT denotes retention time.

In summary, the LC/ESI-MS^2^ employed for the structural analysis of GSL-derived oligosaccharides on the porous graphitized carbon column provided a powerful platform that allowed the discrimination of isomeric glycan structures and allowed clear deduction of the carbohydrate sequence based on the typical series of C- and B-type fragment ions obtained by MS^2^ analysis. Moreover, the diagnostic cross-ring ^0,2^A/^0,2^A-H_2_O and ^2,4^A fragment ions of antepenultimate N-GlcNAc distinguished neolacto series (Galβ4GlcNAc) from lacto series (Galβ3GlcNAc) (60). For instance, the presence of fragmentation ions at *m/z* 427/409 (^0,2^A_3_/^0,2^A_3_-H_2_O, Fig. 4, *A* and *B*) allowed the identification of linkage positions, *i.e.*, type 1 or type 2 chain, and indicated that Hex_3_HexNAc_1_Hex_1_ ion at *m/z* 852 was H5-2 rather than H5-1, which is in correlation with the previously published data (56, 60, 61). Furthermore, the characteristic diagnostic ions resulting from the double glycosidic cleavage of 3-linked branches supported the identification of type 1 and type 2 core chains as well as enabled the differentiation of A, B, Le^a^, Le^b^, Le^x^, Le^y^ blood group epitopes. In case of Le^a/b^, the presence was supported by the fragmentation ions at *m/z* 348 (Fig. 4, *C* and *D*), while Le^x/y^ was indicated by the fragmentation ions at *m/z* 364 (Fig. 4, *C* and *F*) and *m/z* 510 (Fig. 4*E*). Neolacto tetrasaccharides (*i.e.*, nLc_4_, Fig. 5*A*) were further elongated (*e.g.*, nLc_6_ in Fig. 5*B*) or capped with blood group epitopes (*e.g.*, A6-2 and B6-2 in Fig. 5, *E* and *F*, respectively). More interestingly, the presence of P1-5 (Fig. 5*C*) and PX2-5 (Fig. 5*D*) was only detected in the pooled tumor tissue. It should also be mentioned that double peak formation was observed in most GSL subclasses (Figs. 2, *A* and *B*, and 6) and is most likely due to the existence of both α and β anomers of glucose at the reducing end. The identical composition of these double peaks was also confirmed by MS^2^ analysis, as illustrated in Figure 7. The α-/β-anomers can be condensed by reduction of the samples. However, when analyzing reduced samples, the predominance of C-type fragment ions that allow a straightforward interpretation of the carbohydrate sequence is lost, and instead, a mixture of B, C, Y, and Z ions is obtained, making interpretation more difficult (55). Overall, a clear distinction between GSL profiles of normal and tumor pancreatic tissues was found. The neutral GSL-derived oligosaccharides identified and structurally characterized by LC/ESI-MS^2^ in the N-GSL fractions obtained from tumor and normal pancreatic tissues of PDAC patients are summarized in Table 2.

**Table 2:** Summary of GSL-derived oligosaccharides of the neutral GSL fractions released by rEGCase II and identified by LC/ESI-MS^2^ in pooled tumor and normal pancreatic tissues of patients with pancreatic ductal adenocarcinoma

Trivial name (Abbreviation)		Oligosaccharide sequence							Pooled tissues		m/z of [M-H]^-^	Retention time∗ [min]			
									T	N		T		N	
PX2 penta	PX2-5			GalNAcβ3	Galβ4	GlcNAcβ3	Galβ4	Glc	+	-	909.3	24.5	---	---	---
P1 penta	P1-5			Galα4	Galβ4	GlcNAcβ3	Galβ4	Glc	+	-	868.3	26.9	---	---	---
Neolactotetra	nLc_4_				Galβ4	GlcNAcβ3	Galβ4	Glc	+	+	706.3	20.9	---	20.7	---
Neolactohexa	nLc_6_		Galβ4	GlcNAcβ3	Galβ4	GlcNAcβ3	Galβ4	Glc	+	+	1071.4	27.0	27.6	26.9	27.6
H type 1 penta	H5-1			Fucα2	Galβ3	GlcNAcβ3	Galβ4	Glc	+	+	852.3	19.4	20.5	19.3	20.4
H type 2 penta	H5-2			Fucα2	Galβ4	GlcNAcβ3	Galβ4	Glc	-	+	852.3	---	---	21.5	22.2
Lewis^a^ penta	Le^a^-5			Galβ3	(Fucα4)	GlcNAcβ3	Galβ4	Glc	+	-	852.3	16.3	16.9	---	---
Lewis^x^ penta	Le^x^-5			Galβ4	(Fucα3)	GlcNAcβ3	Galβ4	Glc	+	+	852.3	16.2	---	16.0	---
Lewis^b^ hexa	Le^b^-6		Fucα2	Galβ3	(Fucα4)	GlcNAcβ3	Galβ4	Glc	+	+	998.3	15.4	16.1	15.2	15.9
Lewis^y^ hexa	Le^y^-6		Fucα2	Galβ4	(Fucα3)	GlcNAcβ3	Galβ4	Glc	+	+	998.3	17.9	---	17.7	---
Lewis^x^ hepta	Le^x^-7	Galβ4	(Fucα3)	GlcNAcβ3	Galβ4	GlcNAcβ3	Galβ4	Glc	+	-	1217.4	22.5	23.0	---	---
A type 2 hexa	A6-2		GalNAcα3	(Fucα2)	Galβ4	GlcNAcβ3	Galβ4	Glc	-	+	1055.3	---	---	16.0	16.8
B type 2 hexa	B6-2		Galα3	(Fucα2)	Galβ4	GlcNAcβ3	Galβ4	Glc	+	+	1014.3	16.4	17.3	16.3	17.1
B type 2 hepta	B7-2	Galα3	(Fucα2)	Galβ4	(Fucα3)	GlcNAcβ3	Galβ4	Glc	+	+	1160.3	14.1	---	13.9	---

A + sign stands for ′detected′, while a - sign stands for ′not detected′. T denotes tumor tissue and N denotes normal tissue.

∗ if two retention times are stated, it means that these oligosaccharides chains appeared as double peaks.

#### LC/ESI-MS^2^ of native GSL

Native total N-GSL and A-GSL fractions isolated from human normal and tumor pancreatic tissues of PDAC patients were separated by hydrophilic interaction liquid chromatography (HILIC) and subsequently analyzed by LC/ESI-MS^2^ coupled with a capillary HILIC column in the negative ion mode, detected mostly as [M–H]^-^. The quality of HILIC runs was poor, even when rerunning the samples, and the intensity of the signal was generally low, which complicated the identification of GSL in the samples. The sensitivity issues caused that LC/ESI-MS and LC/ESI-MS^2^ analyses of the total fraction of GSL of human pancreatic tissues did not provide too much information, which resulted in the identification of only a few species of GSL in human pancreatic tissues. The nomenclature and shorthand notation of individual lipid species follow the standardized system for reporting lipid structures, as described by Liebisch *et al.* (62).

##### Total N-GSL fractions

To obtain an overview of the ceramide composition of the native N-GSL fractions from the pooled pancreatic tissues, these fractions were analyzed by LC/ESI-MS^2^ using a HILIC column. This yielded very weak MS spectra that, together with subsequent MS^2^ analysis, allowed the reliable identification of only a few GSL species. Among these N-GSL were nLc_4_ and Gb_4_ with 18:1;O2/16:0 ceramide (*m/z* 1225.8), together with H type 2 and Lewis^a/x^ pentaosylceramides with 18:1;O2/16:0 ceramide (*m/z* 1371.8) and 18:1;O2/16:0;O ceramide (*m/z* 1387.8).

##### Total A-GSL fractions

**Figure 8:**
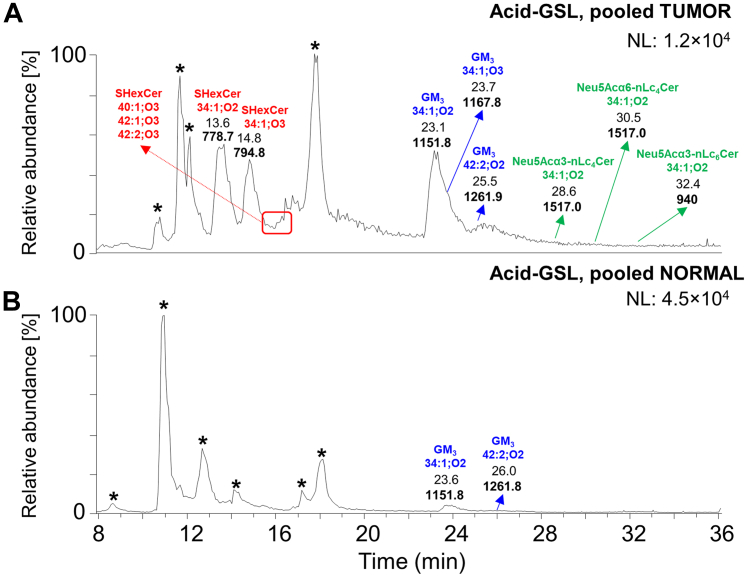
**LC/ESI-MS characterization of the acid glycosphingolipid fractions of human pancreas isolated from pooled tumor and normal pancreatic tissues.** Base peak chromatograms (BPCs) from LC/ESI-MS (negative ion mode at *m/z* 500–1300, retention time 8–36 min) of the acid glycosphingolipid fractions obtained from pooled (*A*) tumor pancreatic tissues and (*B*) normal pancreatic tissue. The identification was based on the retention times, determined molecular masses, and subsequent MS^2^ sequencing. The glycosphingolipids identified in the chromatograms were as follows: sulfatides (SHexCer) with 34:1;O2, 34:1;O3, 40:1;O3, 42:1;O3, and 42:2;O3 ceramides, monosialodihexosylgangliosides (GM_3_) with 34:1;O2, 34:1;O3, and 42:2;O2 ceramides, and monosialylated neolactotetrasylceramides and neolactohexaosylceramides (*i.e.*, Neu5Ac-nLc_4_Cer and Neu5Ac-nLc_6_Cer, respectively) with 34:1;O2 ceramides. NL denotes the normalization level (*i.e.*, intensity of the most abundant peak), and ∗ denotes nonglycolipid contaminants.

Figure 8 illustrates the BPC of total A-GSL fractions of pooled tumor (Fig. 8*A*) and normal (Fig. 8*B*) tissues. The pooled tumor sample contained dominant sulfatides and gangliosides, while the former ones were not detected in the pooled normal tissue (Fig. 8, *A* and *B*). Trace amount of other acidic GSL was also detected in pooled tumor tissues.

**Figure 9:**
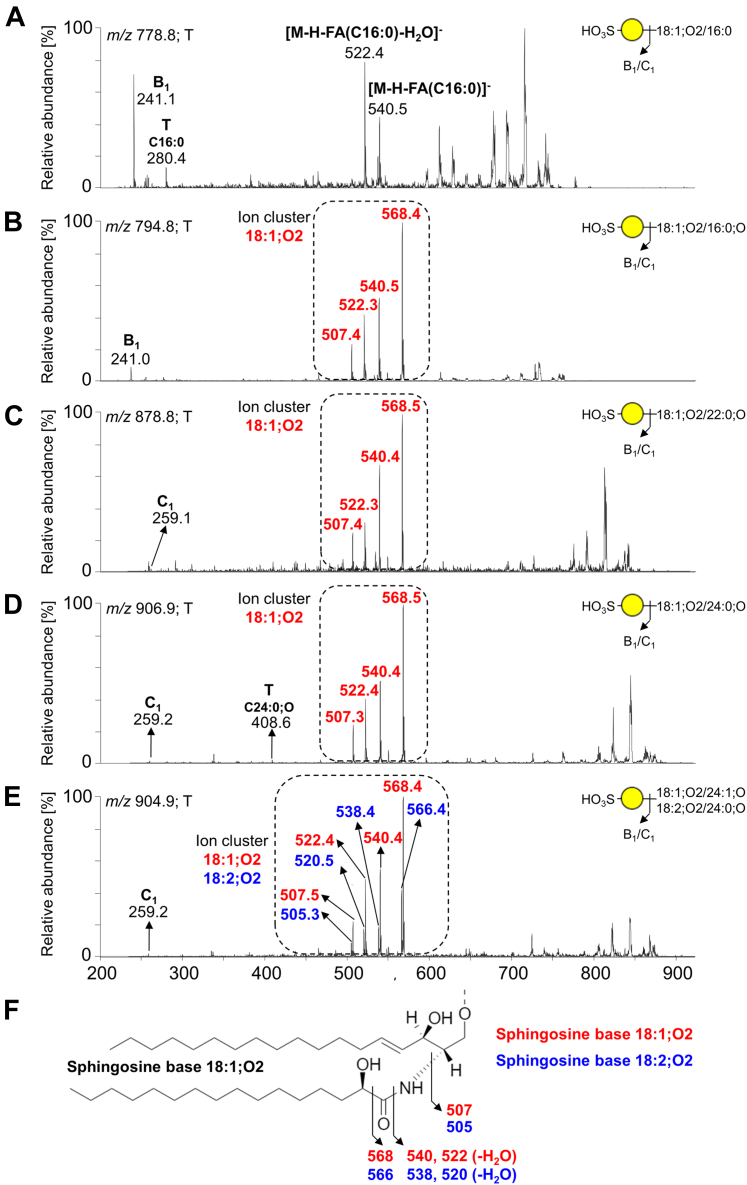
**MS**^**2**^**spectra of sulfatides from the acid glycosphingolipid fraction of pooled tumor pancreatic tissues with the respective interpretation formulas.** MS^2^ spectrum of deprotonated molecule at (*A*) *m/z* 778.8 at retention time 13.6 min (SHexCer 18:1;O2/16:0), (*B*) *m/z* 794.8 at retention time 14.8 min (SHexCer 18:1;O2/16:0;O), (*C*) *m/z* 878.8 (SHexCer 18:1;O2/22:0;O), (*D*) *m/z* 906.9 (SHexCer 18:1;O2/24:0;O), (*E*) *m/z* 904.9 (SHexCer 42:2;O3), and (*F*) demonstration of the ion cluster formation for hydroxylated sulfatides. The identification of the glycosphingolipid species was based on their retention times, determined molecular masses, and subsequent MS^2^ sequencing. T denotes tumor tissue.

The presence of sulfatides was indicated by B_1_ ions at *m/z* 241.1 or C_1_ ions at *m/z* 259.1, demonstrating a terminal SO_3_-Hex in their MS^2^ spectra (Fig. 9). The BPC obtained from LC-ESI/MS of the A-GSL fraction from pooled tumor tissues (Fig. 8*A*) gave two major ions of deprotonated molecules at *m/z* 778.8 and *m/z* 794.8. MS^2^ of these ions identified sulfatides (SO_3_-3Galβ1Cer) with 18:1;O2/16:0 (Figs. 9*A*) and 18:1;O2/16:0;O (Fig. 9*B*) ceramides, respectively. In addition, MS^2^ of three other minor ions with retention times around 16 min (Fig. 8*A*) suggested characteristic spectra for sulfatides with 18:1;O2/22:0;O ceramide (*m/z* 878.8), 18:1;O2/24:0;O ceramide (*m/z* 904.8), and 18:1;O2/24:1;O together with 18:2;O2/24:0;O (both *m/z* 906.8) ceramide (see Fig. 9, *C*–*E*). The presence of sulfatide species with sphingosine base 18:1;O2 and 18:2;O2 together with an α-hydroxy–fatty acyl (FA) substituent were confirmed by a specific ion cluster formation (Fig. 9*F*) at *m/z* 568.4, 540.5, and 522.3 (typical for sphingosine bases 18:1;O2) and at *m/z* 566.4, 538.4, and 520.5 (typical for sphingosine bases 18:2;O2). In contrast, nonhydroxylated sulfatides lack this ion cluster and are represented by fragment ions corresponding to the loss of FA from the deprotonated molecule (*i.e.*, [M-H]^-^-FA) that is commonly accompanied by the additional loss of water (*i.e.*, [M-H-H_2_O]-FA). The clear distinction between MS^2^ spectra of nonhydroxylated and hydroxylated sulfatides is well illustrated in the previous works of Yuki *et al.* (63) and Hsu and Turk (64, 65).

**Figure 10:**
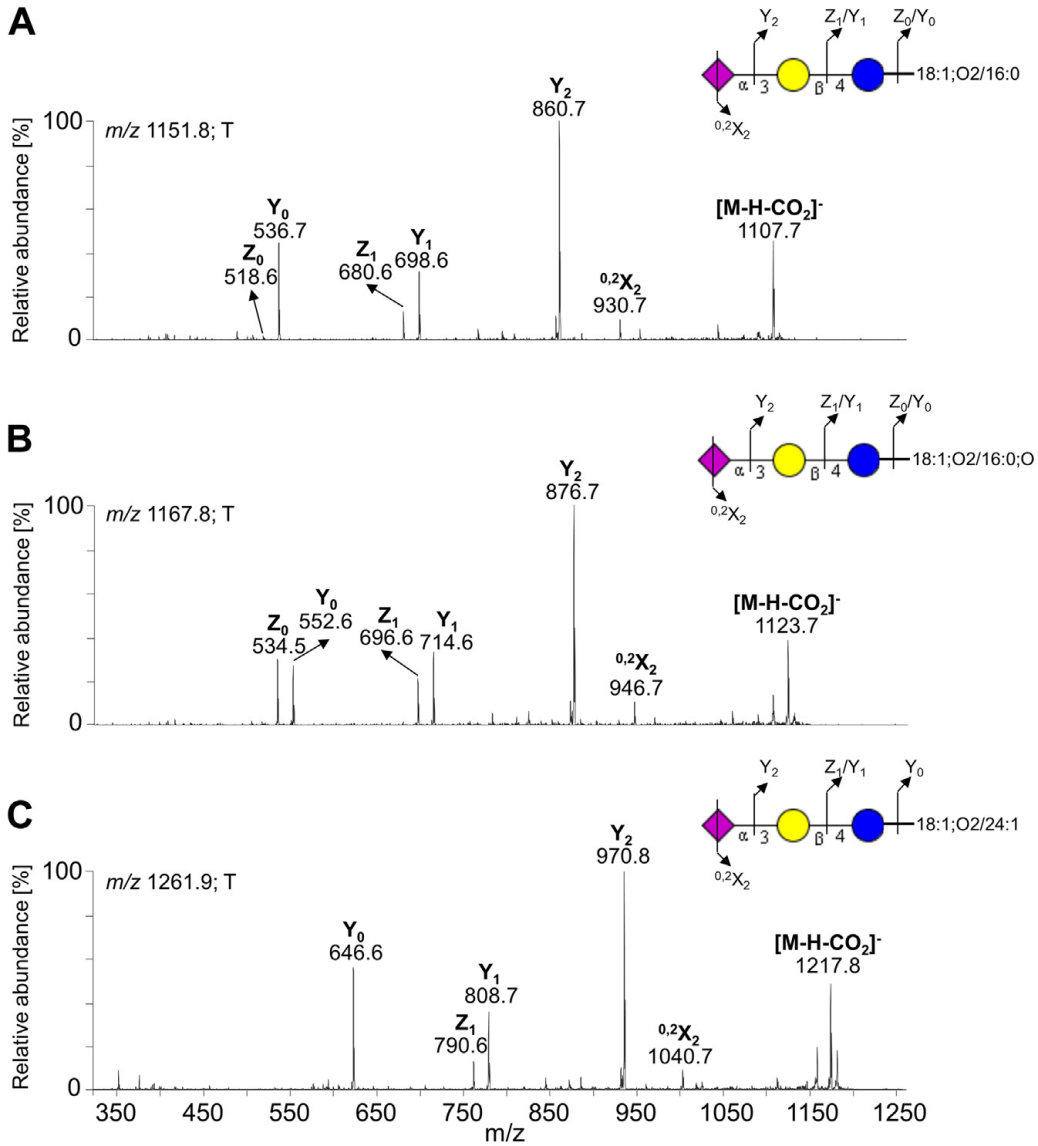
**MS**^**2**^**spectra of monosialodihexosylgangliosides (GM**_**3**_**) from the acid glycosphingolipid fraction of pooled tumor pancreatic tissues with the respective interpretation formulas.** MS^2^ spectrum of deprotonated molecule at (*A*) *m/z* 1151.8 at retention time 23.1 min (GM_3_ 18:1;O2/16:0), (*B*) *m/z* 1167.8 at retention time 23.7 min (GM_3_ 18:1;O2/16:0;O), and (*C*) *m/z* 1261.9 at retention time 25.5 min (GM_3_ 18:1;O2/24:1). The identification of the glycosphingolipid species was based on their retention times, determined molecular masses, and subsequent MS^2^ sequencing. T denotes tumor tissue.

The gangliosides were detected in both pooled tumor and normal tissues. One of the major deprotonated ions from pooled tumor tissues was observed at *m/z* 1151.8 (Fig. 8*A*). The MS^2^ spectrum of this ion yielded a series of Y/Z ions (*i.e.*, Y_0_ at *m/z* 536.7, Z_0_ at *m/z* 518.5, Y_1_ at *m/z* 698.6, Z_1_ at *m/z* 680.6, and Y_2_ at *m/z* 860.6), which implies an oligosaccharide with the composition of NeuAc_1_Hex_2_ (Fig. 10*A*). Moreover, there was ^0,2^X_2_ fragment ion at *m/z* 930.7 arising from the cross-ring cleavage of the terminal NeuAc and [M-H-CO_2_]^-^ ion at *m/z* 1107.7, resulting from the loss of CO_2_ from the corresponding deprotonated molecule. Taken together, these features indicated a ganglioside with carbohydrate sequence of Neu5Ac-Hex-Hex with ceramide 18:1;O2/16:0 (*i.e.*, GM_3_ 34:1;O2). MS^2^ of two other deprotonated ions at *m/z* 1167.8 and 1261.9 also indicated the presence of GM_3_ gangliosides, namely, GM_3_ 18:1;O2/16:0;O and GM_3_ 18:1;O2/24:1 (Fig. 10, *B* and *C*), respectively.

**Figure 11:**
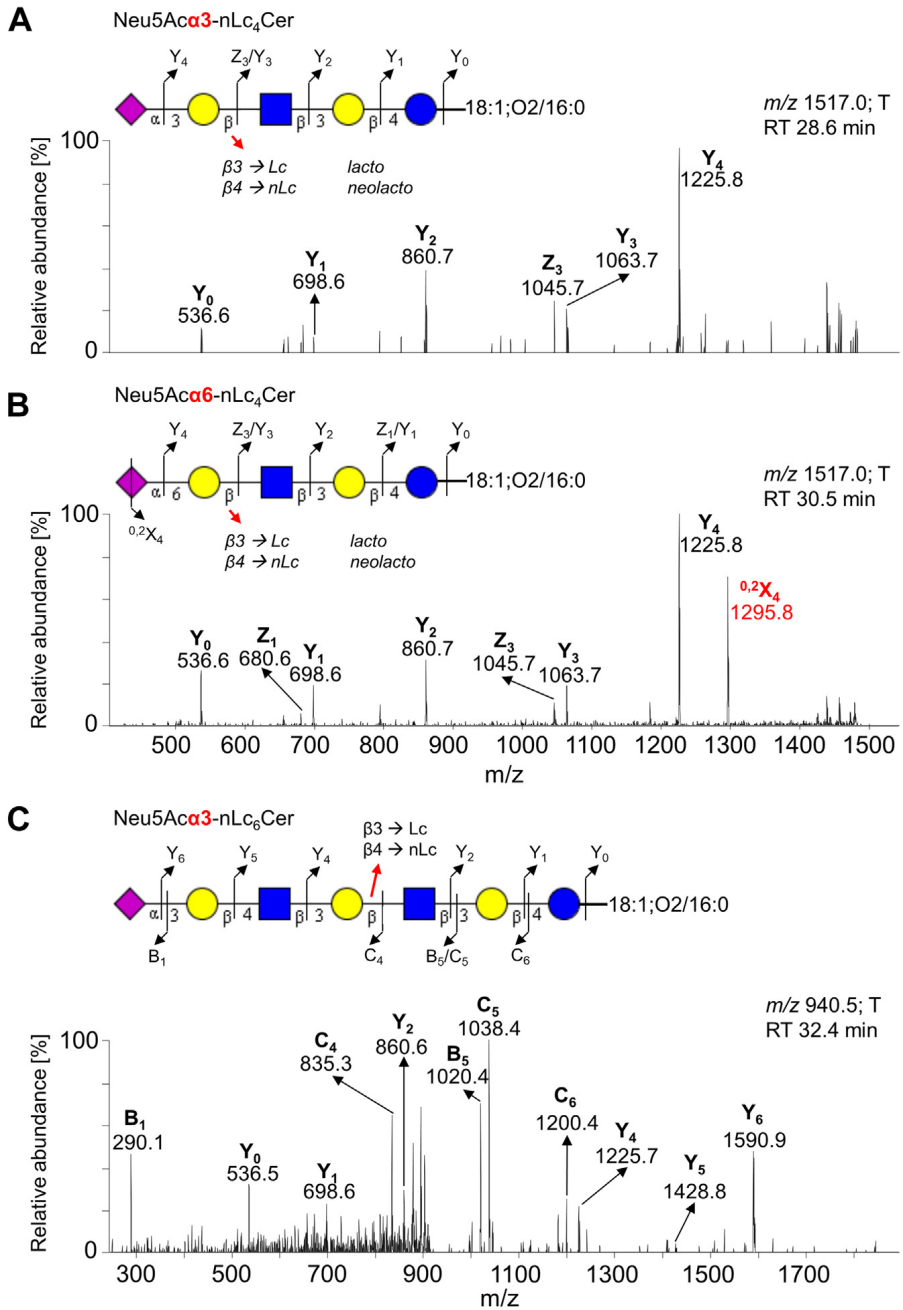
**MS**^**2**^**spectra of sialylated neolactotetrasylceramides and sialylated neolactohexaosylceramides (Neu5Ac-nLc**_**4**_**Cer/nLc**_**6**_**Cer) from the acid glycosphingolipid fraction of pooled tumor pancreatic tissues with the respective interpretation formulas**. MS^2^ spectrum of deprotonated molecule at (*A*) *m/z* 1517.0 at retention time 28.6 min (Neu5Acα3-nLc_4_Cer 18:1;O2/16:0), (*B*) *m/z* 1517.0 at retention time 30.5 min (Neu5Acα6-nLc_4_Cer 18:1;O2/16:0), and (*C*) *m/z* 940.5 (doubly charged ion) at retention time 32.4 min (Neu5Ac-nLc_6_Cer 18:1;O2/16:0). The identification of the glycosphingolipid species was based on their retention times, determined molecular masses, and subsequent MS^2^ sequencing. T denotes tumor tissue.

Few other minor acidic GSL were also detected. The minor ion at *m/z* 1517.0 corresponds to a monosialylated neolactotetraosylceramide *(i.e.*, Neu5Ac-nLc_4_Cer), as characterized by MS^2^ sequencing (Fig. 11). The glycan sequence was deduced from a series of Y-/Z-type fragment ions (*i.e.*, Y_0_ at *m/z* 536.6, Y_1_ at *m/z* 698.6, Y_2_ at *m/z* 860.7, Y_3_ at *m/z* 1063.7, Y_4_ at *m/z* 1225.8, Z_1_ at *m/z* 680.6, and Z_3_ at *m/z* 1045.7). In addition, we found that the ion at *m/z* 1517.0 represents two GSL structures. These two GSL species were distinguished based on distinct retention times and the specific ^0,2^X_4_ fragment ion at *m/z* 1295.8 arising from the cross-ring cleavage of sialic acid. This fragment ion is highly abundant (>50 % of relative intensity) in α6-linked sialic acid, whereas it is low abundant or absent in α3-linked sialic acid (66). Collectively, these features were recognized as Neu5Acα3-nLc_4_Cer (eluting at 28.6 min, Fig. 11*A*) and Neu5Acα6-nLc_4_Cer (eluting at 30.5 min, Fig. 11*B*) with ceramide 18:1;O2/16:0. Neu5Acα3-nLc_4_Cer and Neu5Acα6-nLc_4_Cer are termed as iso-CD75s- and CD75s-ganglioside, which elevate in pancreatic tumor (67).

Another minor doubly charged ion (*i.e.*, [M-2H]^2-^) at *m/z* 940.5, which corresponded to a singly charged ion at *m/z* 1882.0, was found at 32.4 min in LC/ESI-MS of A-GSL fraction from pooled tumor tissues. MS^2^ of this ion (Fig. 11*C*) identified a monosialylated (neo)lactohexaosylceramide, *i.e.*, Neu5Acα3-(n)Lc_6_ with 18:1;O2/16:0 ceramide.

The BPC of the total A-GSL fraction from pooled normal pancreatic tissues was very weak, and we found only gangliosides (Fig. 8*B*). The main ions observed (Fig. S1) correspond to the GM_3_ ganglioside with 18:1;O2/16:0 ceramide (*m/z* 1151.8, see MS^2^ spectrum in Fig. S1*A*) and 18:1;O2/24:1 ceramide (*m/z* 1261.8, see MS^2^ spectrum in Fig. S1*B*). The observed fragmentation patterns of sialylated GSL were consistent with the work published by Chai *et al.* (68).

### Chromatogram-binding assay

**Figure 12:**
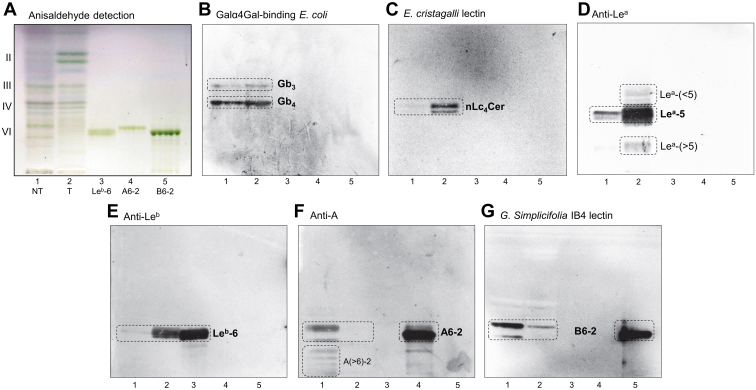
**Binding of antibodies and lectins to subfractions of neutral glycosphingolipids obtained from pooled human pancreatic ductal adenocarcinoma tissues and surrounding normal tissues.** Thin-layer chromatogram with anisaldehyde detection (*A*) and autoradiograms obtained by binding Galα4Gal-recognizing P-fimbriated *Escherichia coli* strain 291-15 (*B*), Galβ4GlcNAc-recognizing *Erythrina cristagalli* lectin (*C*), monoclonal antibodies directed against the blood group Le^a^ (*D*), Le^b^ (*E*), and A (*F*) determinants, and terminal Galα-recognizing *Griffonia simplicifolia* IB4 lectin (*G*). The separation of glycosphingolipids and subsequent chromatogram-binding assays were performed as described in “Experimental procedures”. Lanes: lane 1, neutral glycosphingolipid fraction of pooled normal pancreatic tissue (NT), 40 μg; lane 2, neutral glycosphingolipid fraction of pooled pancreatic ductal adenocarcinoma tissue (T), 40 μg; lane 3, reference Le^b^ hexosylceramide (Le^b^-6, Fucα2Galβ3(Fucα4)GlcNAcβ3Galβ4Glcβ1Cer), 4 μg; lane 4, reference A type 2 hexosylceramide (A6-2, GalNAcα3(Fucα2)Galβ4GlcNAcβ3Galβ4Glcβ1Cer), 4 μg; lane 5, reference B type 2 hexosylceramide (B6-2, Galα3(Fucα2)Galβ4GlcNAcβ3Galβ4Glcβ1Cer), 4 μg. The Roman numbers to the left of chart A denote the number of carbohydrate units in the bands.

**Figure 13:**
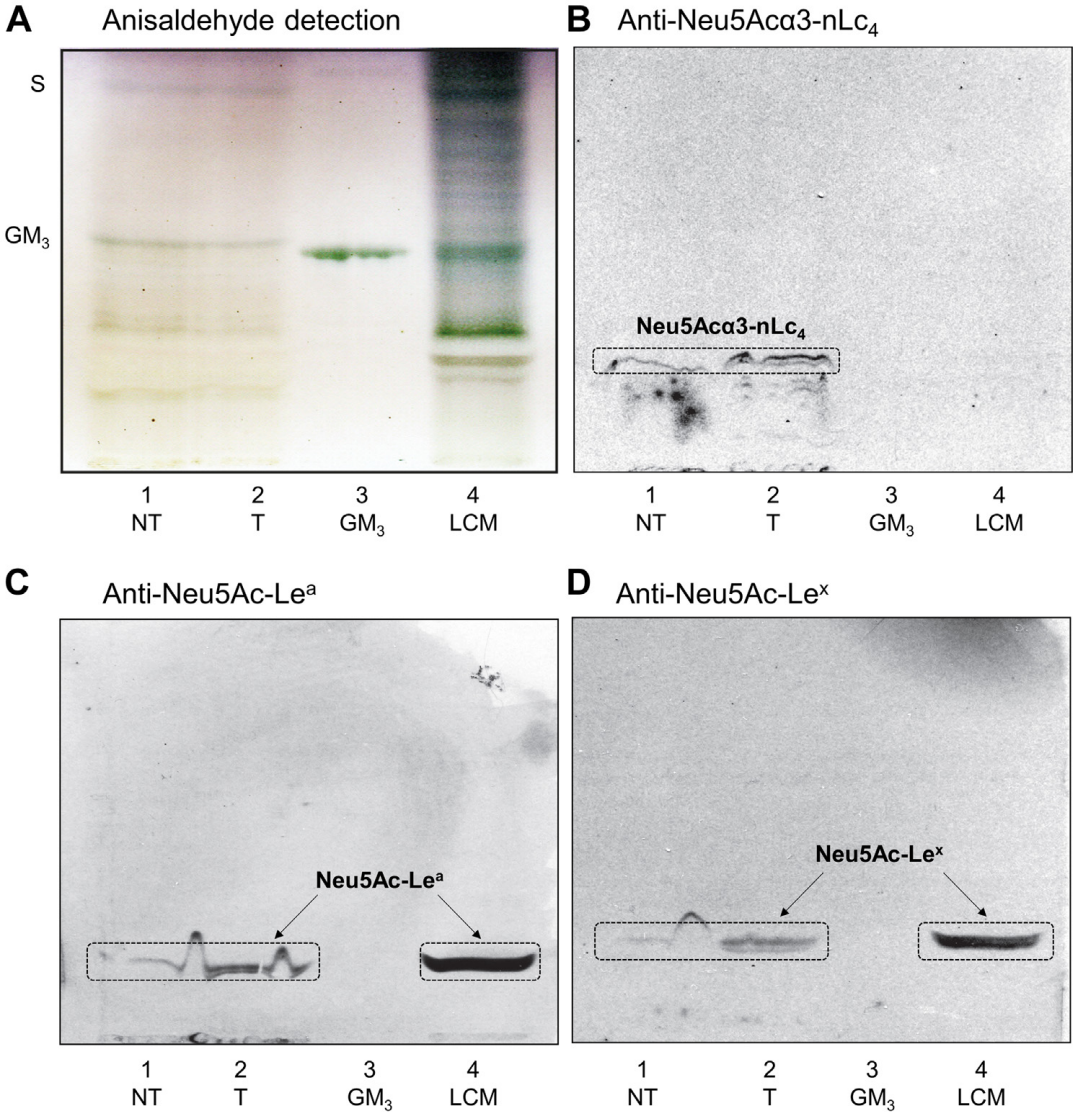
**Binding of antibodies to subfractions of acid glycosphingolipids obtained from pooled human pancreatic ductal adenocarcinoma tissues and surrounding normal tissues.** Thin-layer chromatogram with anisaldehyde detection (*A*) and autoradiograms obtained by binding of monoclonal antibodies directed against the Neu5Acα3-nLc_4_ (*B*), Neu5Ac-Le^a^ (*C*), and Neu5Ac-Le^x^ (*D*) determinants. The separation of glycosphingolipids and subsequent chromatogram-binding assays were performed as described in “Experimental procedures”. Lanes: lane 1, acid glycosphingolipid fraction of pooled normal pancreatic tissue (NT), 40 μg; lane 2, acid glycosphingolipid fraction of pooled pancreatic ductal adenocarcinoma tissue (T), 40 μg; lane 3, reference Neu5Ac-GM_3_, 4 μg; lane 4, reference acid glycosphingolipid fraction of lung cancer metastasis (LCM), 40 μg. The designation S to the left of the chart A indicates the migration level of sulfatides (SO_3_-3Galβ1Cer) and GM_3_ indicates the migration level of the Neu5Ac-GM_3_ gangliosides (Neu5Acα3Galβ4Glcβ1Cer).

Next, the binding of antibodies, lectins, and bacteria to GSL fractions isolated from pooled tumor and normal pancreatic tissues was tested to substantiate the data obtained from LC/ESI-MS^2^. The results of binding assays clearly illustrate the differences in GSL expression in normal and tumor pancreatic tissues (Figs. 12 and 13).

#### Thin-layer chromatography

TLC with anisaldehyde detection of N-GSL fractions showed that the major bands migrated in the monoglycosylceramide to tetraglycosylceramide regions along with some minor slow-migrating compounds (exemplified by pooled normal and tumor pancreatic tissue in Figure 12*A*, lanes 1 and 2). TLC with detection of the resorcinol reagent of A-GSL fractions had several weak bands that confirmed the presence of neuraminic acid and/or its derivatives. Moreover, the TLC with anisaldehyde detection of A-GSL fractions showed the presence of Neu5Ac-GM_3_ in both normal and tumor pancreatic tissue (Fig. 13*A*, lanes 1 and 2), as indicated by comigration with the reference Neu5Ac-GM_3_ (Fig. 13*A*, lane 3). Furthermore, several other slow-migrating and Neu5Ac-containing GSL were found (Fig. 13*A*, lanes 1 and 2). The appearance of double bands on the TLC chromatogram (Fig. 12*A*, lanes 1 and 2) is caused by ceramide heterogeneity.

#### Chromatogram-binding assay for N-GSL fractions

The binding of antibodies, lectins, and bacteria to N-GSL fractions is illustrated in Figure 12. The presence of globotriaosylceramide (Gb_3_) and globotetraosylceramide (Gb_4_) in both pooled normal and tumor tissues were demonstrated by the binding of ^35^S-labeled Galα4Gal-recognizing P-fimbriated *Escherichia coli* strain 291-15 in the triglycosylceramide and tetraglycosylceramide regions (Fig. 12*B*, lanes 1 and 2). This result is consistent with the study published by Distler *et al.* (69).

Next, the Galβ4GlcNAc-binding lectin *of Erythrina crista-galli* provided a more intense staining in the pooled pancreatic tumor tissue fraction (Fig. 12*C*, lane 2) than the pooled normal tissue fraction (Fig. 12*C*, lane 1), which corresponds to higher amounts of neolactotetraosylceramides (nLc_4_Cer) found in pooled tumor tissue.

Furthermore, monoclonal antibodies directed against Le^a^ (Fig. 12*D*) and Le^b^ (Fig. 12*E*) determinants were mainly bound to the fractions obtained from pooled tumor tissues (Fig. 12, *D* and *E*, lane 2), which confirmed the higher amounts of Le^a^ pentosylceramide (Le^a^-5) and Le^b^ hexosylceramide (Le^b^-6) detected by LC/ESI-MS^2^ in the tumors. A considerably weaker binding of anti-Le^a^ and anti-Le^b^ antibodies was also observed in pooled normal tissues (Fig. 12, *D* and *E*, lane 1). Additionally, some compounds that migrate above and below the pentasaccharide region were recognized by anti-Le^a^ antibodies (Fig. 12*D*, lane 2) indicating the presence of more complex GSL with the Le^a^ epitope in the tumor tissue.

In contrast, the monoclonal antibodies directed against the blood group A determinants (Fig. 12*F*) and the *Griffonia simplicifolia* IB4 lectin recognizing Galα terminals, *i.e.*, binding to blood group B determinants (Fig. 12*G*), were bound mainly to the fraction obtained from pooled normal pancreas tissues (Fig. 12, *F* and *G*, lane 1). A weak binding of *G. simplicifolia* IB4 lectin was observed indicating the presence of determinants of blood group B in the fraction obtained from pooled tumor tissues (Fig. 12*G*, lane 2), while no binding of anti-A antibodies was observed in pooled tumor tissues (Fig. 12*F*, lane 2). Additionally, several other compounds migrating below the hexasaccharide region were recognized by anti-A antibodies and indicate more complex GSL with blood group A determinants (Fig. 12*F*, lane 1) in the pooled normal pancreas tissues. Taken together, these results support the hypothesis that GSL with the determinants of blood groups A and B were predominantly present in the fraction obtained from pooled normal pancreas tissues.

A well-known problem with carbohydrate-binding ligands is that many of them are not as specific as it is claimed (70, 71, 72). They may show cross-reactive binding to other glycans or in some cases be nonbinding. However, the binding specificities of the ligands used in this study have been well characterized by us and others (71, 73, 74) and used in many previous studies.

#### Chromatogram-binding assay for A-GSL fractions

The binding of antibodies to the A-GSL fractions is illustrated in Figure 13. The antibodies directed against Neu5Acα3-nLc_4_ determinants were bound to both pooled normal and tumor pancreatic tissue fractions (Fig. 13*B*, lanes 1 and 2), confirming the presence of sialyl-nLc_4_. No binding of anti-Neu5Acα3-Lc_4_ was observed. Additionally, anti-Neu5Ac-Le^a^ (Fig. 13*C*) and anti-Neu5Ac-Le^x^ (Fig. 13*D*) antibodies were mainly bound to the fraction obtained from pooled tumor pancreatic tissue (Fig. 13, *C* and *D*, lanes 1 and 2), indicating higher amounts of sialyl-Le^a^ (sLe^a^) and sialyl-Le^x^ (sLe^x^) pentaosylceramides. The former one is also known as carbohydrate antigen 19-9 (CA 19-9), which is known as a pancreatic cancer marker suitable for the monitoring of disease progress but not suitable for early cancer detection. The presence of sLe^a^ and sLe^x^ in the fractions was also indicated by comigration with the reference A-GSL fraction obtained from lung cancer metastatic tissue (Fig. 13, *C* and *D*, lane 4) since it has previously been shown that these sialylated GSL play a role in lung cancer (75, 76). In line with this, a higher amount of Le^a^-5 pentaosylceramides was also detected by LC/ESI-MS^2^ analysis in tumor tissue (Table 2).

## Discussion

The present work is a systematic and detailed investigation of mainly neutral GSL and further acid GSL isolated from human pancreatic tissues of patients with PDAC. The identification and structural characterization are accomplished with a combination of TLC, chemical staining, binding of carbohydrate-recognizing ligands (antibodies, lectins, and bacteria), and LC/ESI-MS^2^, with a major focus on complex GSL.

GSL patterns of pooled human pancreatic tissues revealed that glycan profiles of tumor pancreatic and surrounding normal pancreatic tissues differ in the region from four to seven sugar units. The lipid and glycan profiling investigated here revealed that the major N-GSL of tumor pancreatic tissues identified by LC/ESI-MS^2^ were GSL with the blood group Le^a^ and Le^b^ determinants together with neolactotetraosylceramides (nLc_4_Cer) (Fig. 2*B*), while the predominant components of normal tissues were GSL with the blood group A and B determinants (Fig. 2*A*). These findings are remarkable since the type 2 core chain of complex GSL was dominating in human normal pancreatic tissues compared to the complex GSL in human pancreatic cancer tissues, where type 1 core chain was mainly found. These results are also supported by the virtually identical results obtained with the binding assay, as illustrated in Figure 12. Furthermore, we found GSL with the blood group Le^x^, Le^y^, and H determinants and neolactohexaosylceramides (nLc_6_Cer) in both pooled normal and tumor pancreatic tissues. Moreover, PX2 and P1 pentaosylceramides alongside Le^x^ heptosylceramides were characterized as minor components in pooled tumor pancreatic tissues. Additionally, the presence of globotriaosylceramides (Gb_3_) and globotetraosylceramides (Gb_4_) in both pooled samples was indicated by the binding assay (see Fig. 12*B*), although these were not identified and characterized by LC/ESI-MS^2^. The absence of globo-series GSL in MS spectra may be in line with the relative resistance of globo-series GSL to hydrolysis by rEGCase II, as previously reported (28, 55, 77, 78).

In case of A-GSL fractions, we obtained very little information from both pooled tissue samples, since the MS spectra did not allow the identification of a larger number of GSL. Nevertheless, several sulfatides and GM_3_ gangliosides were identified and characterized as the main components of the pooled tumor tissues together with other minor compounds such as monosialylated neolacto(tetra/hexa)osylceramides (Neu5Ac-nLc_4_Cer/nLc_6_Cer) (Fig. 8*A*). Sulfatides and GM_3_ gangliosides with 34:1;O2 and 34:1;O3 ceramides were the most predominant GSL species observed (Figs. 9, *A* and *B* and 10, *A* and *B*). Additionally, Neu5Ac-Le^a^ (*i.e.*, sLe^a^ or also CA 19-9 biomarker) and Neu5Ac-Le^x^ GSL were identified by binding assay as well, despite not being characterized by mass spectrometry.

Importantly, the results presented in this report support that alterations in GSL composition, including aberrant glycosylation, sialylation, and/or fucosylation, are an integral part of malignant transformation and tumor progression (6, 22, 27, 32, 46, 75, 79, 80, 81, 82). Interestingly, striking differences in fucosylation, representing one of the most important oligosaccharide modifications linked to cancer, have been previously reported in cell lines (81, 83) and tumor tissues (79, 80) and therefore appear to be a promising target for cancer diagnosis and therapy (84). The changes in glycan structures in PDAC are linked to the expression of glycosyltransferases and related to the formation of Lewis blood group antigens. Deregulations of fucosyltransferases (FUTs) in PDAC have previously been reported (85). Specifically, FUT1 preferentially fucosylate type 2 core chains, while FUT2 and FUT3 prioritize type 1 chains as a substrate (86). Here, we demonstrate that there is a predominance of fucosylated type 1 core GSL (*i.e.*, Le^a^-5 and Le^b^-6) and nLc_4_Cer in pancreatic tumors, whereas the major compounds in the nontumor tissues are blood A and B GSL (*i.e.*, A6-2, B6-2, and B7-2) on type 2 core chains. Thus, the overexpression of Lewis blood group antigens Le^a^ and Le^b^ in PDAC may be associated with the upregulation of FUT2 and/or FUT3. Furthermore, the higher amount of nLc_4_Cer *i.e.*, type 2 chain) in PDAC tissues may be due to the downregulation of FUT1, which by adding a Fuc to the terminal Gal of nLc_4_Cer creates a H type 2 determinant, which is the precursor for the subsequent action of a GalNAcT and a GalT creating the blood group A and B determinants. Clearly, further studies are needed to clarify these results. We should also note that the relative amounts of GSL in the N-GSL fractions (Fig. 6) were different between tumor and normal pancreatic tissues.

Furthermore, GSL with blood group A and B determinants are declined or practically eliminated compared to normal tissues of the same patient where they predominate. We can only speculate that individuals carrying blood groups A and B determinants may be more prone to develop pancreatic cancer based on the comparison of tissue samples, which is in agreement with previously published studies (87, 88, 89, 90). To our knowledge, there is only one previous study of GSL in normal human pancreas published by Breimer (91) in 1984, where the occurrence of both type 1 and type 2 core chain blood group ABH and Lewis glycolipids in pancreas is reported in two individuals with blood group A and B. However, more studies will be needed to clarify the value of these findings. The present work focuses on qualitative analysis and lipid profiling of mainly complex GSL in human pancreatic cancer, which are not commonly included in conventional lipidomic methods and extends the coverage of GSL commonly analyzed in cancer research. Therefore, future studies should also investigate whether the differences observed between normal and pancreatic tumor tissues translate into differences in GSL profile between PDAC patients and healthy subjects.

## Experimental procedures

### Chemicals and solvents

Methanol (*p.a.*) was purchased from VWR International AB. Ethanol (>99.5%) was purchased from Solveco AB. Toluene (HPLC grade, >99.8%) was purchased from RCI Labscan. Chloroform (≥99.8%), lithium chloride (≥99%), potassium hydroxide, sulfuric acid (95–97%), acetic acid glacial (100%), dichloromethane (anhydrous, stabilized with amylene, ≥99.8%), acetic anhydride (*p.a.*, >99.5%), pyridine (>99.5%), anisaldehyde (4-methoxybenzaldehyde, for synthesis), and resorcinol were purchased from Sigma-Aldrich, Merck KGaA (Darmstadt, Germany). Silica gel S (particle size: 32–63 μm, 230–400 mesh ASTM) were purchased from Riedel-de Haën. DEAE-cellulose 23 was purchased from Whatman. Polyisobutylmethacrylate was purchased from Sigma-Aldrich. Deionized water (Milli Q) was prepared with Purelab Flex 2 water purification system (AB Ninolab) and all organic solvents were redistilled prior to use.

### Reference GSL

N-GSL and A-GSL fractions were isolated as described by Karlsson (92). Individual GSL were isolated by repeated chromatography on silicic acid columns and by HPLC and further identified and characterized by mass spectrometry (55, 93) and proton NMR spectroscopy (94).

### Sample collection

Tissue samples including tumor and surrounding normal pancreatic tissues were obtained from 12 different patients with PDAC (see Table 3). The samples were collected at the University Hospital Olomouc and kept in a freezer at −80 °C prior to further processing. The study was approved by the Regional Ethics Committee of University Hospital Olomouc, Czech Republic (reference number 57/15) following the Declaration of Helsinki and the General Data Protection Regulations. All patients received written and verbal information before signing an informed consent for inclusion in the study. The complete list of samples with clinicopathological information is described in “Table S1” in Supporting information. The information on blood groups is not available.

**Table 3:** Initial amounts of the tissue samples (*i.e.*, before lyophilization) used for the isolation of GSL

Sample no.	Initial amount of the tissue sample [mg]											
	673	682	690	694	705	711	758	778	796	800	840	845
Pa-T	84	60	88	210	49	145	90	65	53	96	92	57
	Total pooled Pa-T sample → 1.089 g											
Pa-N	112	226	114	165	110	130	99	515	242	69	171	139
	Total pooled Pa-N sample → 2.092 g											

Pa-T denotes pancreatic tumor tissue, Pa-N denotes pancreatic normal tissue, NA denotes not applicable.

### Isolation and preparation of GSL

Samples obtained from 12 PDAC patients were pooled separately for tumor and adjacent nontumor tissues, and lyophilized. The nontumor tissue is further annotated as “normal tissue”. The initial amounts of the tissue samples (*i.e.*, before lyophilization) used for the isolation of GSL are listed in Table 3.

Due to the limited amount of starting material that restricted the experiments performed, we used the micro method described by Barone *et al.* (48), which is based on the method originally introduced by Prof. Karlsson, for the isolation of total N-GSL and A-GSL. The only modification was the use of Soxhlet extraction at the beginning of the experiment. The scheme of the procedure used for the preparation of total N-GSL and A-GSL is shown in Figure 1, and a detailed description of the protocol is described in “Protocol S1” in Supporting information. The obtained total GSL fractions (*i.e.*, N-GSL and A-GSL) were characterized by a combination of TLC, binding of carbohydrate-recognizing ligands in chromatogram-binding assays, and LC/ESI-MS^2^ as described below.

### Thin-layer chromatography

TLC was performed continuously throughout the whole extraction protocol to control each step of the procedure. The TLC was accomplished on aluminum-backed or glass-backed silica gel 60 high performance TLC plates (Merck). GSL mixtures (40–80 μg) and/or pure GSL (4 μg) were applied to high performance TLC plates and chromatographed with a solvent system composed of CHCl_3_/MeOH/H_2_O (60:35:8, v/v/v). The developed plates were air-dried and subsequently chemically detected using the anisaldehyde staining reagent for both GSL fractions (*i.e.*, anisaldehyde/acetic acid/H_2_SO_4_ in proportions 1:98:2, v/v/v) (72) or the resorcinol staining reagent (95, 96) for total A-GSL fractions (*i.e.*, 0.2 g of resorcinol dissolved in HCl/0.1 M CuSO_4_/H_2_O in proportions 80:0.25:19.75, v/v/v).

### Chromatogram-binding assays

Binding of monoclonal antibodies to GSL separated on thin-layer chromatograms was performed as described by Barone *et al.* (48, 72). A detailed description of the binding procedure is described in “Protocol S2” in the Supporting information. The binding of ^35^S-labeled Galα4Gal-binding P-fimbriated *E. coli*, ^125^I-labeled *E. crista-galli* lectin, *G. simplicifolia* lectin IB4, and anti-Neu5Ac-nLc_4_/Lc_4_ to GSL in thin-layer chromatograms was performed as previously reported (73, 74, 97, 98). The specifications of carbohydrate-recognizing ligands tested for binding to the GSL of human PDAC tissues are listed in Table 4.

**Table 4:** Carbohydrate-binding ligands used in chromatogram-binding assays

Antibodies	Clone/ Designation	Manufacturer/ Reference	Dilution	Isotype	Binding specificity
Anti-blood group A	HE-193	GeneTex/Abcam	1:500	IgM	GalNAcα3(Fucα2)Gal
Anti-Lewis^a^	7LE	GeneTex/Abcam	1:100	IgG	Galβ3(Fucα4)GlcNAc
Anti-Lewis^b^	T218	Santa Cruz Biotechnology	1:200	IgM	Fucα2Galβ3(Fucα4)GlcNAc
Anti-Neu5Ac-nLc_4_^c^	LM1:1a	(98)	1:500	IgM	Neu5Acα3Galβ4GlcNAc
Anti-Neu5Ac-Lc_4_^c^	TR4/SL-50	(98)	1:500	IgM	Neu5Acα3Galβ3GlcNAc
Anti-Neu5Ac-Le^a^	116-NS-19-9	Signet	1:50	IgG1	Neu5Acα3Galβ3(Fucα4)GlcNAc
Anti-Neu5Ac-Le^x^	KM93	Merck	1:50	IgM	Neu5Acα3Galβ4(Fucα3)GlcNAc
P-fimbriated *Escherichia coli*	Strain 291-15	(74)	−	−	Galα4Gal
*Erythrina crista-galli* lectin	−	Vector Laboratories Inc.	1:100	−	Galβ4GlcNAc
*Griffonia simplicifolia* lectin IB4	−	Advanced Targeting System	1:200	−	Galα

c This antibody was a kind gift of Dr. Maria Blomqvist, Institute of Biomedicine, Department of Clinical Chemistry and Transfusion Medicine, Sahlgrenska Academy, University of Gothenburg, Göteborg, Sweden.

### Endoglycoceramidase digestion

rEGCase II from *R.* spp. (Takara Bio Europe S.A.) was used for the digestion of N-GSL as described (57). A detailed description of the whole procedure is listed in “Protocol S3” in Supporting information. The neutral oligosaccharides released from GSL were resuspended in 50 μl of deionized water prior to analysis.

### LC/ESI-MS^2^ of native and GSL-derived oligosaccharides

The native N-GSL and A-GSL fractions (50 μg) dissolved in 30 μl of MeOH/MeCN (3:1, v/v) and GSL-derived oligosaccharides dissolved in 50 μl of deionized water were injected (3 μl) using a PAL HTC-xt autosampler (CTC Analytics AG) with a 2 μl sample loop. Native N-GSL and A-GSL were separated on a HILIC capillary column (100 × 0.25 mm) packed in-house with 5 μm polyamine II particles (YMC Europe GmbH), and GSL-derived neutral oligosaccharides were separated on a porous graphitized carbon capillary column (100 × 0.25 mm) packed in-house with 5 μm porous graphite particles (Hypercarb, Thermo-Hypersil) as described (57).

Detailed descriptions of the LC/ESI-MS^2^ conditions for the analysis of native GSL and GSL-derived oligosaccharides are listed in the Supporting information in “Methods S1” and “S2”, respectively.

### Data processing

**Figure 14:**
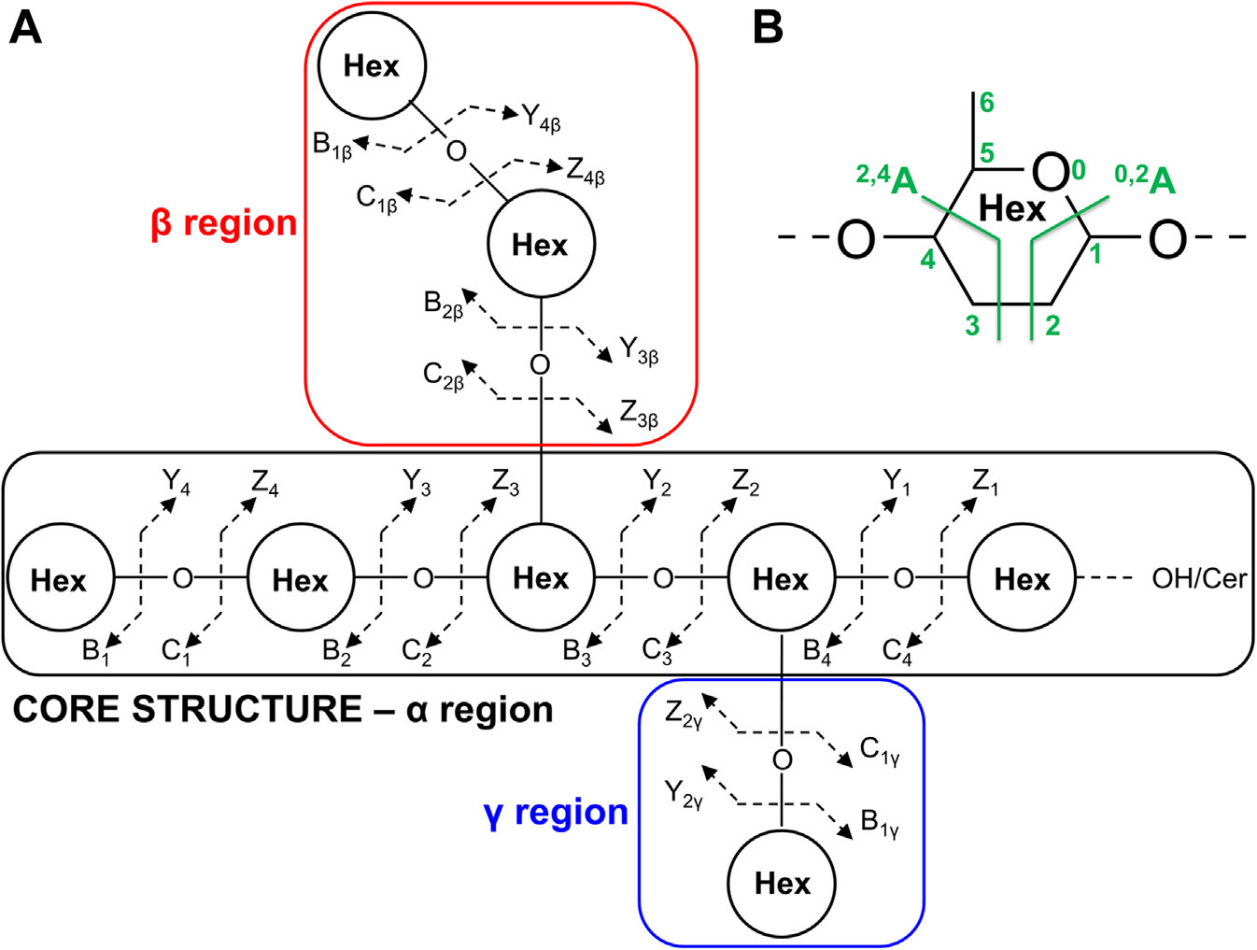
**Nomenclature and typical fragmentation patterns for cleavages of linear and branched oligosaccharides, adopted and modified from** (100)**.***A*, nomenclature and fragmentation of linear and branched oligosaccharides. *B*, nomenclature and description of the cross-ring cleavages within the monosaccharide unit.

Thermo Scientific Xcalibur software (Version 2.0.7) was used for data processing. Assignment of the glycan sequence and GSL structures was done manually based on the knowledge of mammalian biosynthetic pathways together with the help of the GlycoWorkbench tool (Version 2.1, https://glycoworkbench.software.informer.com/download/) (99), Lipid Maps MS analysis tools (https://www.lipidmaps.org/tools/ms/). The characteristic fragmentation patterns of the identified GSL subclasses follow general rules and nomenclature for the cleavages of linear and branched oligosaccharides (100) (see Fig. 14) and have previously been well described (56, 61, 63, 64, 65, 68). Structures were verified by comparison of retention times and in-depth examination of relevant MS^2^/MS^3^ spectra of GSL or GSL-derived oligosaccharides from reference GSL (55).

## Data availability

Raw data were uploaded to Glycopost (https://glycopost.glycosmos.org/preview/1308639356629738236b971), password 7924, accessed on 01, 01 2023.

## Supporting information

This article contains supporting information.

## Conflict of interest

The authors declare that they have no conflicts of interest with the content of this article.
