# Supplementary material for: Comprehensive characterization of complex glycosphingolipids in human pancreatic cancer tissues
Source: J Biol Chem. 2023 Jan 19;299(3):102923. doi: 10.1016/j.jbc.2023.102923 (PMC9976472; doi:10.1016/j.jbc.2023.102923)
Supplement: Supporting information [file mmc1.pdf]

SUPPORTING INFORMATION

Comprehensive characterization of complex glycosphingolipids in human pancreatic cancer tissues

Karel Hořejší[1,2], Chunsheng Jin[3], Zuzana Vaňková[1], Robert Jirásko[1], Ondřej Strouhal[4], Bohuslav Melichar[4], Susann Teneberg[5,*], and Michal Holčapek[1,*]

[1] University of Pardubice, Faculty of Chemical Technology, Department of Analytical Chemistry, Studentská 573, 532 10, Pardubice, Czech Republic

[2] University of South Bohemia in České Budějovice, Faculty of Science, Department of Chemistry, Branišovská 1760, 370 05, České Budějovice, Czech Republic

[3] University of Gothenburg, Sahlgrenska Academy, Proteomics Core Facility, S-405 30 Göteborg, Sweden

[4] Palacký University Olomouc, Faculty of Medicine and Dentistry and University Hospital, Department of Oncology, I. P. Pavlova 6, 775 20, Olomouc, Czech Republic

[5] University of Gothenburg, Sahlgrenska Academy, Institute of Biomedicine, Department of Medical Biochemistry and Cell Biology, P.O.Box 440, S-413 90 Göteborg, Sweden

* **Corresponding Authors Information:**

Michal Holčapek, E-mail: michal.holcapek@upce.cz, Phone: +420 466 037 087,

ORCID: 0000-0003-3978-1249

Susann Teneberg, E-mail: susann.teneberg@medkem.gu.se, Phone: +46 31-786 34 92,

ORCID: 0000-0003-1957-9553

Running title: Characterization of glycosphingolipids in pancreatic cancer

Figure S1: MS^2 spectra of monosialodihexosylgangliosides (GM_3) from the acid glycosphingolipid fraction of pooled normal pancreatic tissues with the respective interpretation formulas.

Table S1: Clinicopathological information of the patients included in this study.

Protocol S1: Detailed protocol for the isolation of GSL.

Protocol S2: Detailed protocol for the chromatogram binding assays.

Protocol S3: Detailed protocol for the endoglycoceramidase digestion procedure.

Method S1: LC/ESI-MS^2 conditions for the analysis of native GSL

Method S2: LC/ESI-MS^2 conditions for the analysis of GSL-derived oligosaccharides

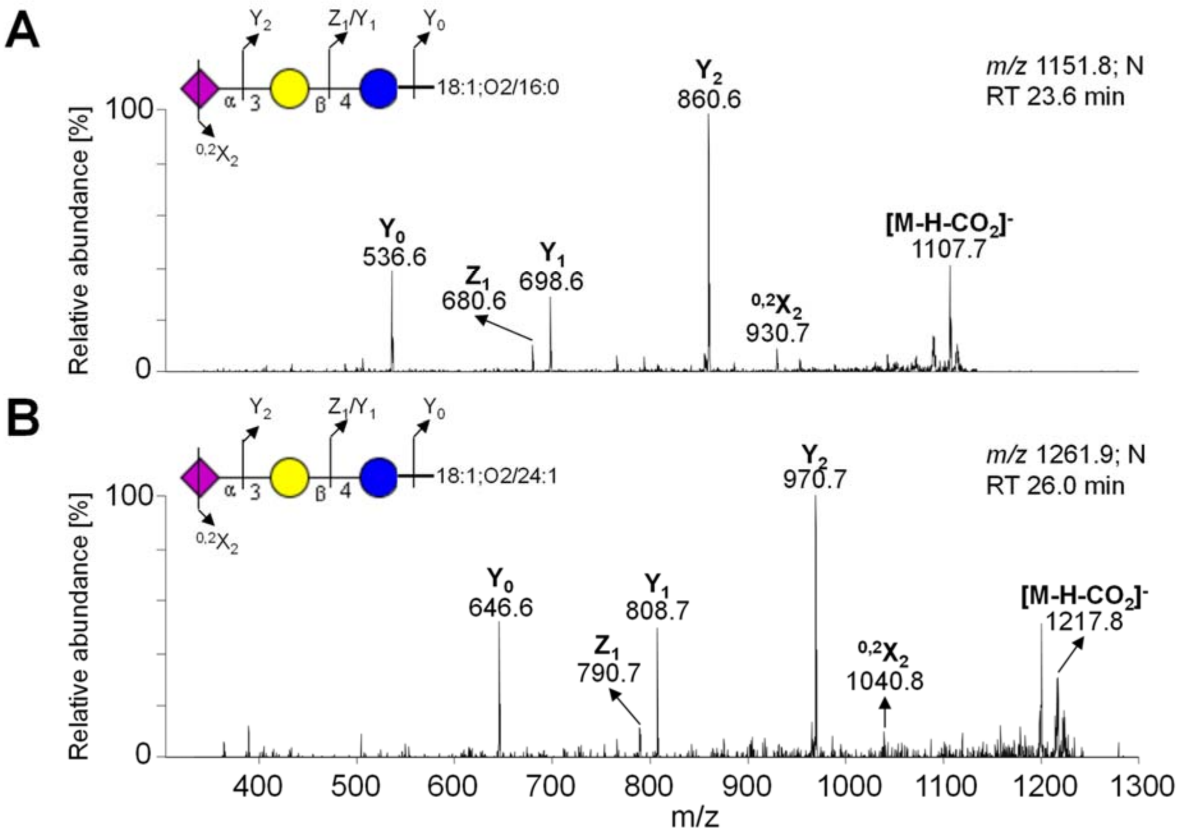

Figure S1. MS^2 spectra of monosialodihexosylgangliosides (GM$_3$) from the acid glycosphingolipid fraction of pooled normal pancreatic tissues with the respective interpretation formulas. MS^2 spectrum of deprotonated molecule at **(A)** *m/z* 1151.8 at retention time 23.6 min (GM$_3$ 18:1;O2/16:0), and **(B)** *m/z* 1261.9 at retention time 26.0 min (GM$_3$ 18:1;O2/24:1). The identification of the glycosphingolipid species was based on their retention times, determined molecular masses, and subsequent MS^2 sequencing. N denotes normal tissue.

Table S1

Clinicopathological information of the patients included in this study.

Sample No.	Gender	Year of birth	Height [cm]	Weight [kg]	BMI	Tumor characteristics		
						Type	Grade	TNM
**673**	Male	1951	166	74	26.9	Ductal adenocarcinoma	G2	T3N2M0
**682**	Female	1945	154	53	22.3	Ductal adenocarcinoma	G1	T3N0
**690**	Male	1946	182	99	29.9	Ductal adenocarcinoma	G3	T2N2Mx
**694**	Female	1948	163	90	33.9	Ductal adenocarcinoma	G1-2	T2M0Mx
**705**	Female	1955	156	48	19.7	Ductal adenocarcinoma	G2-3	T3NxM0
**711**	Female	1955	165	48	17.6	Ductal adenocarcinoma	G2	T2N1M0
**758**	Female	1948	165	65	23.9	Ductal adenocarcinoma	G2	T3N2M0
**778**	Male	1943	171	72	24.6	Ductal adenocarcinoma	G2	T1N1M0
**796**	Male	1957	172	83	28.1	Ductal adenocarcinoma	G2	T1N2Mx
**800**	Male	1957	176	78	25.2	Ductal adenocarcinoma	G1	T2N2M0
**840**	Female	1977	161	65	25.1	Ductal adenocarcinoma	G3	T2N0M0
**845**	Female	1957	168	80	28.3	Ductal adenocarcinoma	G2	T2N1M0

Protocol S1

Detailed protocol for the isolation of GSL.

The pooled lyophilized tumor (0.606 g) and normal (1.232 g) pancreatic tissues were processed separately. The extraction was performed in two steps in a Soxhlet apparatus with 700 ml of $CHCl_3$/MeOH in a ratio of 2:1 (v/v) followed by 1:9 (v/v) for 24 hours and using boiling chips granules (2–8 mm, Merck) to prevent superheating. The resulting extracts were pooled and evaporated to dryness under the N_2 stream using a 60 ° C water bath. All extract residues were subjected to mild alkaline hydrolysis by adding 1 ml of 0.2M KOH to MeOH. The mixture was briefly sonicated, vortexed for 1 min, and left to react at room temperature in the dark for 3 hours. The hydrolyzed extract was dialyzed against running tap water for 3 days. After dialysis, the sample was dried, and the resulting residue was acetylated by adding 1.5 ml of $CHCl_3$/pyridine/acetic anhydride (1:1:1, v/v/v) and left to react at room temperature in the dark overnight. The acetylation was stopped by adding 3 ml of MeOH followed by 5 ml of toluene. After drying, the sample was subjected to silicic acid chromatography, eluted with CH_2Cl_2 (fraction 1), 5% methanol in chloroform (MiC) + 10% MiC + 15% MiC (fraction 2), and finally 75% MiC + MeOH (fraction 3). All fractions were dried. The fraction containing GSL (*i.e.*, fraction 2) was deacetylated by the addition of 1 ml of 0.2M KOH in MeOH/MeOH/toluene (2:1:1, v/v/v) at room temperature in the dark for 30 minutes. The deacetylated sample was dialyzed and after drying subjected to ion-exchange chromatography using diethylaminoethyl cellulose as a sorbent (DEAE-cellulose). The sample was transferred to a chromatography column packed with DEAE-cellulose and soaked in $CHCl_3$/MeOH (2:1, v/v) and left to equilibrate overnight. Finally, two fractions were eluted using $CHCl_3$/MeOH (2:1, v/v) and MeOH (fraction 1; N-GSL) and 5% (w/v) LiCl in MeOH (fraction 2; A-GSL). Fraction 2 was dialyzed for 3 to 5 days.

Protocol S2

Detailed protocol for the chromatogram binding assays.

Thin-layer chromatograms with separated GSL were dipped in a mixture of 0.5% (w/v) polyisobutylmethacrylate in diethylether/n-hexane (5:1, v/v) for 1 minute, and then freely air-dried for 15 minutes. Subsequently, the plates were placed separately in Petri dishes and soaked in phosphate buffered saline (PBS, pH 7.3) containing 2% (w/v) bovine serum albumin (BSA), 0.1% (w/v) NaN_3 and 0.1% (w/v) Tween 20 (PBS/BSA/Tween 20) for 2 hours at room temperature to reduce background and nonspecific bindings. The plates were then carefully covered with a monoclonal antibody suspension diluted in PBS/BSA/Tween 20 (the dilution used for each monoclonal antibody is given in **Table 4**) and incubated at 4 °C overnight. Plates were then washed four times with PBS and treated with secondary antibodies. Two types of secondary antibodies were used for detection. The first type was alkaline phosphate-conjugated goat anti-mouse polyvalent IgG/IgA/IgM antibodies (Sigma-Aldrich; A0162) diluted 1:500 and alkaline phosphate-conjugated goat anti-human monovalent IgM antibodies (Sigma-Aldrich; A3437) diluted 1:400 in PBS/BSA/Tween 20. Visualization was performed by covering the plates with SigmaFast BCIP/NBT (5-bromo-4-chloro-3-indolyl phosphate/nitro blue tetrazolium) solution. The other type of secondary antibodies used was ^{125}I-labeled rabbit anti-mouse antibodies labeled by 1,3,4,6-tetrachloro-3α,6α-diphenyl-glycoluril (Pierce iodination reagent, 28600; Thermo Fisher Scientific) using the IODO-GEN method according to the manufacturer´s instructions and diluted to 2×10^6 cpm/ml in PBS/BSA/Tween 20. Plates were incubated at room temperature for 2 hours, then washed six times with PBS, air-dried, and autoradiographed for 12–24 hours using XAR-5 X-ray films (Carestream, 8941114).

Protocol S3

Detailed protocol for the endoglycoceramidase digestion procedure.

A total of 50 µg of total N-GSL were resuspended in 100 µl of 50 mM sodium acetate buffer (pH 5.0) containing 120 µg of sodium cholate *(i.e.,* ionic detergent) and briefly sonicated. Thereafter, 10 mU of rEGCase II was added, and the mixture was incubated at 37°C for 48 hours. Following the incubation, the reaction was stopped by the addition of $CHCl_3$/MeOH/H_2O to the final proportion 8:4:3 (v/v/v), vortexed for 1 min and allowed to partition overnight. The upper phase containing oligosaccharides was transferred to a glass test tube, dried under a stream of N_2 and reconstituted in 30 µl of deionized water. The extract thus obtained was separated from detergent and salts using a solid-phase extraction on a Sep-Pak Accell Plus QMA cartridge (sorbent bed: 360 mg, particle size: 37–55 µm, pore size: 300 Å, volume: 0.8 ml; Waters, Milford, MA, USA) containing a silica-based strong anion-exchanger. Firstly, the QMA cartridge was conditioned with 2 × 0.8 ml of MeOH and equilibrated with 2 × 0.8 ml of H_2O. Afterwards, the extract (30 µl) was loaded onto the cartridge and two drops were expelled to a fresh test tube using air. Then, the original glass test tube was rinsed with 50 µl of H_2O, briefly vortexed, and the resulting solution was loaded onto the cartridge. The elution was done with 0.8 ml of H_2O and the eluate was collected in the test tube containing two drops expelled during the sample loading. Finally, any remaining solvent in the cartridge was purged with air, collected in the same test tube as the eluate, and the resulting solution was evaporated to dryness under the stream of N_2.

Method S1

LC/ESI-MS^2 conditions for the analysis of native GSL

The separation was carried out using an Accela 600 binary pump (Thermo Fisher Scientific, San José, CA, USA) that delivered a flow of a mobile phase of 0.25 ml/min, which was split in an 1/16" microvolume-T (0.15 mm bore) (Vici AG International, Schenkon, Switzerland) by a 50 cm × 50 µm i.d. fused silica capillary before the injector of the autosampler, allowing approximately 3–5 µl/min through the column. The elution of native N-GSL and A-GSL was carried out with a mobile phase gradient composed of acetonitrile (phase A, MF-A) and 10mM NH_4HCO_3 (phase B, MF-B). The gradient elution program was set as follows: 0 min (100% of MF-B), 40 min (50% of MF-B), 40.01 min (20% of MF-B), 50 min (20% of MF-B), 51 min (100% of MF-B), and 60 min (100% of MF-A). The total run time including the analysis time (40 min), washing step (10 min), and the equilibration of the column (10 min) was 60 min. Both native GSL fractions were analyzed in negative ion mode on a three-segment LTQ XL linear quadrupole ion trap mass spectrometer (Thermo Electron, Waltham, MA, USA) equipped with the Ion Max standard ESI source to which the separated oligosaccharides were transferred using a transfer line constructed of a fused silica capillary (30 cm × 50 µm i.d.). The automatic gain control (AGC) was used. ESI settings were set as follows: spray voltage -2.6 kV, ion transfer capillary temperature 275°C, ion transfer capillary voltage -23 kV, and compressed air was used as nebulizer gas (sheath gas flow, 28 arb.; auxiliary gas flow, 1 arb.). Full MS spectra were acquired by scanning the mass range *m/z* 600–2000 (2 microscans with maximum injection time 50 and 100 ms, AGC target value of 30,000), followed by data-dependent MS^2 scans of the three most abundant ions in each scan (2 microscans with maximum injection time 50 and 100 ms, AGC target value of 10,000). The threshold for MS^2 was set to 400 counts. The normalized collision energy was 35%, isolation width *m/z* 3.0 (*m/z* 1.0 for full MS), activation Q = 0.250, activation time of 30 ms, and normal scanning mode (*i.e.*, 16,667 Da/s) was used. The following setup of ion optics system and linear quadrupole ion trap was used for MS, MS^2, and MS^3 analysis: tube lens voltage -98.72 V, skimmer offset 0 V, multipole RF amplifier 400 Vpp, multipole MP00 offset 6.50 V, lens L0 voltage 6.50 V, multipole MP0 offset 6.75 V, lens L1 voltage 12.0 V, gate lens offset 72.0 V, multipole MP1 offset 12.0 V, and front lens voltage 6.75 V.

Method S2

LC/ESI-MS^2 conditions for the analysis of GSL-derived oligosaccharides

The separation was accomplished using an Accela 600 binary pump (Thermo Fisher Scientific, San José, CA, USA) delivering a flow of a mobile phase of 0.25 ml/min, which was splitted in 1/16" microvolume-T (0.15 mm bore) (Vici AG International, Schenkon, Switzerland) by a 50 cm × 50 µm i.d. fused silica capillary before the injector of the autosampler, allowing approximately 3–5 µl/min through the column. The elution of GSL-derived oligosaccharides was performed with the mobile phase gradient composed of 10mM NH_4HCO_3 (phase A, MF-A) and 10mM NH_4HCO_3 in 80% ACN (phase B, MF-B). The gradient elution program was set as follows: 0 min (100% of MF-A), 7 min (95 % of MF-A), 46 min (55% of MF-A), 47 min (0% of MF-A), 54 min (0% of MF-A), 55 min (100% of MF-A), and 75 min (100% of MF-A). The total run time, including analysis time (46 min), the washing step (8 min), and column equilibration (21 min) was 75 min. Separated oligosaccharides were analyzed in negative ion mode on a three-segment LTQ XL linear quadrupole ion trap mass spectrometer (Thermo Electron, Waltham, MA, USA) equipped with the Ion Max standard ESI source to which the separated oligosaccharides were transferred using a transfer line constructed of a fused silica capillary (30 cm × 50 µm i.d.). The automatic gain control (AGC) was used. The ESI settings were as follows: spray voltage -2.6 kV, ion transfer capillary temperature 275°C, ion transfer capillary voltage -37 kV, and compressed air was used as nebulizer gas (sheath gas flow, 26 arb.). Full MS spectra were acquired by scanning the mass range *m/z* 380–2000 (2 microscans with maximum injection time 50 and 100 ms, AGC target value of 30,000), followed by data-dependent MS^2 scans of the three most abundant ions in each scan (2 microscans with maximum injection time 50 and 100 ms, AGC target value of 10,000). The threshold for MS^2 was set to 300 counts. The normalized collision energy was 35%, the isolation width *m/z* 3.0 (*m/z* 1.0 for full MS), activation Q = 0.250, the activation time 30 ms, and the normal scanning mode (*i.e.*, 16,667 Da/s) was used. The following setup of ion optics system and linear quadrupole ion trap was used for MS and MS^2 analysis: tube lens voltage -113.72 V, skimmer offset 0 V, multipole RF amplifier 400 Vpp, multipole MP00 offset 7.50 V, lens L0 voltage 8.50 V, multipole MP0

offset 9.25 V, lens L1 voltage 13.0 V, gate lens offset 60.0 V, multipole MP1 offset 13.50 V, and front lens voltage 10 V.
